# Hypoxia-induced exosomal lncRNA-PVT1 as a biomarker and mediator of EMT in hepatocellular carcinoma

**DOI:** 10.32604/or.2024.056708

**Published:** 2025-05-29

**Authors:** LIBO LIANG, XINYI WANG, YUPING ZENG, HAO CHEN, WEN ZHOU, HONGYING MU, GA LIAO

**Affiliations:** 1 General Practice Medical Center, West China Hospital, Sichuan University, Chengdu, 610041, China; 2 Department of Laboratory Medicine, West China Hospital, Sichuan University, Chengdu, 610041, China; 3 Sichuan Clinical Research Center for Laboratory Medicine, Chengdu, 610041, China; 4 Clinical Laboratory Medicine Research Center of West China Hospital, Chengdu, 610041, China; 5 Medical Big Data Centre, Sichuan University, Chengdu, 610041, China; 6 State Key Laboratory of Oral Diseases & National Center for Stomatology & National Clinical Research Center for Oral Diseases & Department of Information Management & Department of Stomatology Informatics, West China Hospital of Stomatology, Sichuan University, Chengdu, 610041, China

**Keywords:** Hepatocellular carcinoma (HCC), Transcatheter arterial chemoembolization (TACE), Exosome, long noncoding RNA-plasmacytoma variant translocation 1 (lncRNA-PVT1), Epithelial-mesenchymal transformation (EMT)

## Abstract

**Objectives:**

Exosomal long noncoding RNAs (lncRNAs) might facilitate epithelial–mesenchymal transition (EMT) in liver cancer after transarterial chemoembolization (TACE), thereby enhancing tumor cell invasiveness and migration. This study investigated the prognostic role of plasma exosomal long noncoding RNA-plasmacytoma variant translocation 1 (lncRNA-PVT1) in TACE treated hepatocellular carcinoma (HCC).

**Methods:**

Plasma exosomal lncRNA-PVT1 was evaluated via qPCR before and after TACE. Hepatoma cell behavior was investigated in different HCC cell lines. A lncRNA-PVT1 plasmid was synthesized and overexpressed, and si-lncRNA PVT1 was transfected into poorly invasive cells to reveal its influence on cell characteristics. The lncRNA-PVT1–FoxM1 interaction was elucidated using a double-luciferase reporter gene assay. The effect of miRNA-345-5p on minimally invasive hepatoma cells was assessed. Three experimental groups were established: poorly invasive cells, poorly invasive cells co-cultured with exosomes from hypoxia-induced highly invasive cells, and poorly invasive cells co-cultured with normal hepatocyte exosomes. Liver cancer cells were subcutaneously inoculated into nude mice, with subsequent observations of weight, tumor formation, and tumor size.

**Results:**

We identified two lncRNAs (lncRNA-PVT1 and GAPLINC) associated with EMT in the hypoxic microenvironment of liver cancer. Cox multivariate regression analysis was used to establish a prognostic model distinguishing high- and low-risk groups. Hypoxia-induced HepG2 exosomes significantly promoted EMT in poorly invasive HCC cells. LncRNA-PVT1 overexpression and silencing altered E-cadherin, vimentin, and FoxM1 expression, cell proliferation, invasion, migration, and apoptosis. miR-345-5p directly targeted lncRNA-PVT1 and FoxM1, affecting downstream targets. *In vivo*, co-culturing poorly invasive hepatoma cells with exosomes from highly invasive cells increased tumor volumes, upregulated lncRNA-PVT1, FoxM1, Ki67, and MMP9 expression, and downregulated miR-345-5p expression.

**Conclusions:**

Plasma exosomal lncRNA-PVT1 expression is upregulated in highly invasive cells post-hypoxia, potentially serving as a biomarker for evaluating liver cancer prognosis after TACE. Through a miRNA-345-5p-mediated competing endogenous RNA mechanism, it promotes EMT in poorly invasive cells, likely contributing to recurrence and metastasis post-HCC interventional embolization.

## Introduction

Annually, liver cancer accounts for more than 700,000 new cases globally, ranking sixth in incidence and fourth in mortality rates; particularly, among males, it ranks fifth in incidence and second in mortality [1]. Primary liver cancers, with hepatocellular carcinoma (HCC) accounting for more than 85%–90% of cases, are highly vascularized tumors, with 90% of their blood supply originating from the hepatic artery [2]. Apart from surgical removal and liver transplantation, local interventional techniques, including transarterial embolization, drug-eluting bead chemoembolization, and transarterial radioembolization play leading roles in managing liver cancer. Approximately half of patients undergo these treatments during disease progression [3]. For transarterial chemoembolization (TACE), one of the most used minimally invasive surgical treatments for liver cancer, compared to that with conservative treatment, the 2-year survival rate has increased by 23% [4]. However, TACE can intensify the hypoxic environment in remaining tumor cells, leading to epithelial-mesenchymal transition (EMT), a critical process for tumor invasion and metastasis that might influence the long-term success of liver cancer treatments, such as TACE [5]. Hypoxia, a key molecular characteristic of the tumor microenvironment (TME), often affects the metastatic potential of cancer cells by promoting angiogenesis [6]. Increased hypoxia-inducible factor-1α (HIF-1α) affects transcriptional regulation of the oncogene Forkhead box protein M1 (FOXM1), which activates the downstream EMT-related markers N-cadherin and vimentin, leading to their upregulated expression, and has been implicated in promoting EMT [7].

Tumor-derived exosomes play crucial roles in various aspects of tumor progression, including TME remodeling, angiogenesis, invasion, metastasis, and drug resistance [8]. The role of long noncoding RNAs (lncRNAs), which are often more abundant and specifically localized within exosomes than as free-floating molecules, in these processes is increasingly being recognized. Specifically, exosomal lncRNAs might facilitate EMT in liver cancer after TACE, thereby enhancing the invasiveness and migration of remaining cancer cells [9,10]. In this study, the mechanisms underlying EMT following hepatic arterial embolization were investigated, the timing of consolidative treatment was precisely determined, and novel approaches for the post-intervention assessments of tumor invasiveness were explored.

## Methods

### Data source

mRNA and lncRNA expression levels in liver cancer tissues and paracancerous tissues were downloaded from The Cancer Genome Atlas (TCGA) database (https://portal.gdc.cancer.gov) (accessed on 12 November 2024) and the UCSC Xena website (https://xenabrowser.net/datapages/) (accessed on 12 November 2024). The screening criteria were as follows: absolute relative expression fold-change value ≥2 and corrected *p*-value < 0.05. EMT-related genes were downloaded from the dbEMT database (http://www.dbemt.bioinfo-minzhao.org/) (accessed on 12 November 2024) [11], and hypoxia-related genes were obtained from the MSigDB database (https://www.gsea-msigdb.org/gsea/msigdb/index.jsp) (accessed on 12 November 2024) [12]. The online databases Starbase v3.0 [13] and Lncbase v3.0 [14] were employed to screen miRNAs that could bind lncRNA-*PVT1* and the 3′-UTR of *FoxM1* and that exhibited an expression trend opposite to that of lncRNA-*PVT1* and *FoxM1*. These miRNAs were subsequently studied for their involvement in competing endogenous RNA (ceRNA) regulatory mechanisms.

### Participant enrollment

We enrolled patients with HCC who underwent TACE between May and October 2020 and collected pre- and postoperative peripheral blood samples and clinical data. Computed tomography of the abdomen was performed 3–6 months after surgery to observe disease progression. The inclusion and exclusion criteria are listed in Table S1. This study was approved by the Ethics Committee of the West China Hospital, Sichuan University (No. 2018256). Animal experiments were approved by the Animal Ethical and Welfare Committee of Sichuan University in China (No. 20211218A). All the experiments were carried out in accordance with the approved guidelines. The biospecimens used in this study were obtained during previous diagnostic and therapeutic processes, posing minimal risk to the participants. The requirement for informed consent was acknowledgments waived according to the ethical guidelines.

### Identification and isolation of plasma exosomes

Exosomes from plasma were extracted using the Exo Quick™ exosome precipitation solution kit (EXOQ5A-1, System Biosciences Inc., Palo Alto, CA, USA), and those from the cell culture medium were obtained via ultracentrifugation. The exosomes were identified by observing their morphology using transmission electron microscopy, analyzing their size and concentration using nanoflow cytometry, and determining exosomal membrane protein expression using western blotting (WB). For detailed procedures, please refer to the Table S2.

### Cell culture and treatment

Hep G2 (CVCL_AS98, C1110, WheLab, Shanghai, China). Huh7 (CVCL_0336, BNCC337690) and Hep3B (CVCL_1348, BNCC360312, BNCC, Beijing, China) were cultured in Dulbecco’s Modified Eagle’s Medium (DMEM, C11965500BT, GIBCO BRL, CA, USA), supplemented with 10% fetal bovine serum (FBS, 10270-106, California, USA), 100 U/mL penicillin, and 100 µg/mL streptomycin (Pen/Strep, SV30010, Cytiva, Shanghai, China). Cells were maintained at 37°C in a humidified atmosphere containing 5% CO_2_. Media was changed every 2–3 days, and cells were passaged at approximately 80% confluence using trypsin-EDTA (T1300, Solarbio, Beijing, China). All cell lines were routinely tested for mycoplasma contamination using a PCR-based detection method. The tests were conducted every 5 passages to ensure the absence of contamination. When cells reached 90% density, CoCl_2_ (2304895-71-2, Innochem, Shanghai, China) was added for 24 h more to create a hypoxic HCC model. 3-(4,5-Dimethylthiazol-2-yl)-2,5-diphenyltetrazolium bromide (MTT) assay was performed to evaluate cell viability, where Huh7 cells were seeded in 96-well plates at a density of 1000 cells per well. After different treatments, 10 µL of 10 mg/mL MTT solution (298-93-1, Ameresco, Framingham, USA) was added to each well and incubated for 4 h, followed by the addition of 100 µL DMSO to dissolve the formazan crystals. The absorbance was measured at 490 nm via the microplate spectrophotometer (Epoch, BioTek, Winooski, VT, USA). Transwell assays were conducted to assess cell invasion. After digesting the cells and preparing them in serum-free DMEM, 10^5^ cells were added to each Transwell insert pre-coated with Matrigel (40184ES, Yeasen, Shanghai, China). The lower chamber contained DMEM with 20% FBS as a chemoattractant. After 24 h, non-invading cells were removed, and invading cells were fixed, stained with 0.25% crystal violet, and visualized under an inverted microscope (OLYMPUS CKX53, Tokyo, Japan). For the scratch assay, cells were cultured until nearly confluent, and a straight scratch was made using a pipette tip. The cells were washed with PBS to remove debris, and a serum-free medium was added. The wound healing process was monitored by taking images at 0, 24, and 48 h to assess cell migration. Exosomes from various treated hepatocyte cultures were harvested and co-cultured with Huh7 cells. After vortexing, 5 μL of PKH67 labeling dye (PKH67GL, Sigma-Aldrich, St. Louis, MO, USA) was added into 50 μL reaction buffer, followed 50 μL of exosomes. This mixture was incubated at room temperature for 5 min in the dark. For purification, the plunger was removed from the column, and 200 μL of sterilized PBS was added, followed by centrifugation at 50 g for 90 s; the eluate was discarded. The 100 μL labeled exosome preparation was applied to the top of the column and centrifuged at 50 g for 90 s, with the eluate being discarded. The column was then placed into a 1.5 mL centrifuge tube, topped with 200 μL of sterilized PBS, and centrifuged at 50 g for 90 s to collect the eluate. For nuclear staining, a 15 μg/mL DAPI solution (C0060, Solarbio, Beijing, China) was added to the cell culture medium at 1/10th of its volume. Cells were incubated at 37°C for 15 min, followed by two washes with PBS. Fluorescent microscopy images of exosome uptake by Huh7 cells were captured using fluorescence microscopy (OLYMPUS IX73, Tokyo, Japan) at 6 and 12 h.

### Plasmid transfection

To investigate the role of lncRNA-PVT1, we performed overexpression and knockdown experiments using plasmid transfection and siRNA. For overexpression, cells were divided into two groups: one transfected with an empty vector (PCDNA3.1-NC, V790-20, Invitrogen, Carlsbad, CA, USA) and the other with a plasmid encoding lncRNA-PVT1 (PCDNA3.1-LncRNA-PVT1). Cells were seeded and cultured for 24 h to reach approximately 75% confluence. Each transfection involved diluting 1 µg of endotoxin-free plasmid DNA in 400 µL of serum-free medium (DMEM, C11965500BT, GIBCO BRL, California, USA), followed by the addition of 2 µL of Lipofectamine 2000 (11668-019, Invitrogen, Carlsbad, CA, USA). The mixture was gently pipetted to mix thoroughly and then added dropwise to the cells in 4 mL of serum-free medium. Cells were incubated at 37°C with 5% CO_2_ for 6 h, after which the medium was replaced with a 10% FBS complete medium. For the knockdown of lncRNA-PVT1, cells were assigned to two groups: a control group treated with scrambled siRNA (si-NC; AS: 5′-UUCUCCGAACGUGUCACGUdTdT-3′, SS: 5′-ACGUGACACGUUCGGAGAAdTdT-3′) and an experimental group treated with siRNA targeting lncRNA-PVT1 (si-LncRNA-PVT1), for detailed sequences, please refer to the Table S3. The siRNA transfection followed the same protocol as the plasmid transfection, using identical conditions for reagent volumes and incubation.

### Nude mouse xenograft model

4-week-old female BALB/c-nu nude mice were purchased from the Institute of Laboratory Animal Sciences, Chinese Academy of Medical Sciences (Beijing). The initial body weight was 18–22 g per mouse. The mice were randomly divided into three groups, with six mice in each group. The room temperature was maintained at 20°C–22°C with a humidity range of 65%–79% and a 12-h light-dark cycle. The mice had free access to food and water. After 1 week of acclimatization to controlled environmental conditions and regular feeding, Huh7 cells, either untreated or co-cultured with exosomes from hypoxia-treated HepG2 cells, were grown to approximately 90% confluence. The cells were prepared at a concentration of 1 × 10^7^ cells/mL and subsequently injected subcutaneously into the left flanks of mice. were subsequently injected subcutaneously into the left flanks of mice. Visible tumors typically formed subcutaneously within 5 days post-injection, prompting bi-daily measurements of mouse weights and tumor dimensions for 3 weeks, with tumor volume calculated using the formula of $V=\frac{1}{2}\times lenth\times width^{2}$ . After the observation period, the mice were anesthetized and euthanized, while tumors were excised, weighed, and photographed for further analysis.

### Dual-luciferase reporter assay

For dual-luciferase reporter assays, a 1.2 kb region of the Foxm1 promoter was cloned into the pmirGLO vector (E1330, Promega, Madison, WI, USA), which carries the firefly luciferase gene as a reporter. The Renilla luciferase gene, also present in the same vector, served as an internal control. LncRNA-PVT1 and miR-345 were cloned into separate pmirGLO vectors to serve as experimental constructs, the sequences are listed in Table S4. The recombinant plasmids were co-transfected into Huh7 cells using Lipofectamine 2000 (11668-019, Invitrogen, Carlsbad, CA, USA), following the manufacturer’s protocol. Cells were incubated for 48 h at 37°C with 5% CO_2_ before measuring luciferase activity using the microplate luminometer (GloMax 96, Promega, Madison, WI, USA). The luminescence was recorded at a wavelength of 560 nm for firefly luciferase and 480 nm for Renilla luciferase to quantify the regulatory effects of lncRNA-PVT1 on miR-345 and Foxm1 expression.

### Rescue experiment

Huh7 cells were co-cultured with exosomes isolated from hypoxia-treated HepG2 cells under standard conditions at 37°C with 5% CO_2_ for 24 h. Following the co-culture period, the cells were transfected with either miRNA mimics or negative control (NC) mimics (Table S5) using Lipofectamine 2000 (11668-019, Invitrogen, Carlsbad, CA, USA), for detailed sequences, please refer to the Table S5. After an additional 48 h of incubation, the biological functions of the cells were evaluated.

### Real-time quantitative polymerase chain reaction (qRT-PCR)

RNA extracted was conducted with a miRNeasy Serum/Plasma kit (217184, Qiagen, Valencia, CA, USA) and reverse transcription was with the quantinova reverse transcription kit (205411, Qiagen, Valencia, CA, USA) via qRT- PCR instrument (LightCycler 96, Roche, Basel, Switzerland). The compositions were mixed thoroughly and reacted at 25°C for 3 min, 5°C for 10 min, and 85°C for 5 min. Amplification of primer sequences of lncRNA-PVT1 and β-actin with fluorescence quantitative PCR. A QuantiNova SYBR-Green PCR Kit (208054, Qiagen, Valencia, CA, USA) was used for qRT-PCR. The reaction system is presented in Table S6. The compositions were mixed well and put on the machine after transient centrifugation, and the program was set as follows: pre-denaturation at 95°C for 10 min and 45 cycles of denaturation at 95°C for 15 s and annealing at 60°C for 32 s, followed by reaction at 95°C for 10 s, at 65°C for 60 s, and at 97°C for 1 s. Finally, the thermal melting curve was obtained. The present study involved an *in vivo* hypoxic environment. Accordingly, β-actin was used as an internal reference to prevent the influence of hypoxia on the expression of housekeeping genes. The primer sequences of lncRNA-PVT1 and mRNAs are listed in Table S7. The expression of RNAs was calculated with the 2^−ΔΔCt^ method.

### Western blot

In this experiment, cell samples were lysed using RIPA buffer (R0020, Solarbio, Beijing, China) and separated by SDS-PAGE. Protein samples were electrophoresed at 70 V for 30 min and then at 120 V for approximately 1 h. Proteins were transferred to a Polyvinylidene Difluoride (PVDF) membrane (IPVH00010, Millipore, Darmstadt, Germany) at 4°C with a current of 350 mA for 2 h in a wet transfer system. The membrane was blocked with 1×Blotto (NH0001, Leagene, Beijing, China) at room temperature for 2 h, followed by incubation with diluted primary antibodies (dilution ratios are listed in the Table S8) at 4°C overnight. After multiple washes with 1×TBST (PN5211, G-clone, Beijing, China), the membrane was incubated with secondary antibodies (ab7090, abcam, Cambridge, UK) at room temperature for 1.5 h. Finally, western lightning™ chemiluminescence reagent, superkine™ ultra-sensitive ECL substrate (BMU102, Abclonal, Wuhan, China) were applied for detection, and target protein bands were analyzed using a gel imaging system (ChemiDoc XRS+, Bio-Rad, Hercules, PA, USA.).

### Hematoxylin-eosin (HE) and immunohistochemistry (IHC) staining

The embedded 4 μm tissue sections underwent HE staining and IHC. The HE staining followed the manufacturer’s protocol (C0105S, Beyotime, Shanghai, China). Sections were deparaffinized in xylene, rehydrated through descending alcohols to distilled water, stained with Harris hematoxylin for 5 min, differentiated in 0.5% hydrochloric acid ethanol, blued under running water, and counterstained with eosin in 95% ethanol. Dehydration was completed with ascending ethanols and xylene before mounting with neutral resin. Sections were observed and photographed (BX3, Olympus, Tokyo, Japan) at 400x magnification.

Tissue sections were deparaffinized in xylene and rehydrated through graded ethanol to distilled water. Endogenous peroxidase activity was blocked with 3% hydrogen peroxide. Antigen retrieval was performed using citrate buffer at 95°C for 25 min. The sections were incubated in 0.1% Tween 20 in PBS for 10 min at room temperature to perform membrane permeabilization. Sections were blocked, then incubated overnight with primary antibodies (Table S8) at 4°C followed by secondary antibodies (1:300, ab7090, abcam, Cambridge, UK) at room temperature. 3,3′-Diaminobenzidine (DAB, ZLI-9018, ZSGB-bio, Beijing, China) was used for chromogen development, and hematoxylin (C0107, Beyotime, Shanghai, China) was used for counterstaining. Slides were dehydrated, cleared in xylene, and mounted with neutral resin. Staining intensity was evaluated at 200x magnification, and optical density was analyzed using HistoQuest (TissueGnostics, Vienna, Austria).

### Flow cytometry

Cells (10^6^) from 6-well plates were digested with 0.25% trypsin (27250-018, Gibco, Waltham, MA, USA), and centrifuged at 1000 g for 5 min for flow cytometry analysis (Annexin V/PI apoptosis kit, AP101, Multi science, Hangzhou, China). The pellet was resuspended in 195 µL Annexin V-FITC binding buffer, followed by 5 µL Annexin V-FITC stain and 10 µL Propidium Iodide (PI). After mixing, the cells were incubated in the dark for 10 min at room temperature.

### Statistical analysis

Data were analyzed using GraphPad Prism 8.0 software (GraphPad Software, CA, USA). Heat maps of hypoxia and EMT gene expression were constructed using the “pheatmap” package (v 1.0.12) in R (version 4.3.1, R Foundation for Statistical Computing, Vienna, Austria), and differential gene expression analysis was performed with the “limma” package (v 3.58.1). Venn diagrams were generated to identify the intersection of differentially expressed lncRNAs and mRNAs associated with hypoxia and EMT in liver cancer. For statistical evaluation, Student’s *t*-test and Repeated Measures ANOVA were used. For non-normal data, the Wilcoxon rank-sum test and Wilcoxon matched-pairs signed-rank test were applied as appropriate. Univariate and multivariate Cox regression analyses were conducted using the R “survival” package (v 3.5-7), and a LASSO regression model was established using the “glmnet” (v4.1-8) and “survival” (v3.5-7) packages. Model performance was assessed with an ROC curve generated by the “SurvivalROC” package (v 1.0.3.1). Histograms and line charts display the standard error of the median for each experimental group. Bidirectional tests were applied, with a *p*-value of < 0.05 indicating statistical significance. Biological replicates for all experiments numbered at least three.

## Results

### LncRNA- *PVT1* is associated with hypoxia and EMT in liver cancer

General data and preoperative laboratory results of patients with delayed recurrence and early recurrence after surgery are summarized in Table 1. We summarize the demographic information (e.g., age, sex), medical history (e.g., viral hepatitis, metabolic disease), and smoking and drinking history, as well as detailed preoperative laboratory results.

**Table 1:** General data and laboratory preoperative results of patients with delayed recurrence and early recurrence after surgery

	Late recurrence N = 25	Early recurrence N = 27	*p*
Age (years)	59.36 ± 12.64	57.63 ± 12.23	0.618
Sex			
Male	17	21	0.432
Female	8	6	
Medical history			
Viral hepatitis	19	16	0.295
Metabolic disease	0	4	
Alcoholic liver disease	1	1	
Hepatitis B complicated with metabolic disease	1	0	
Smoking history			
Time (years)	0 (0, 20)	2.5 (0,30)	0.655
Number (cigarettes/day)	0 (0, 10)	2.5 (0, 10)	0.556
History of drinking (years)	0 (0,6)	0 (0, 3)	0.493
Family history			
None	23	24	0.707
Cancer	2	3	
First treatment			
Surgery	4	8	0.177
interventional therapy	20	19	
Medication	1	0	
BMI	24.67 ± 8. 97	22.47 ± 8.47	0.09
PIVKA (mAU/mL)	896 (54, 24826)	152 (42, 1451)	0.054
AFP (ng/mL)	29.2 (3.47, 1012)	20.4 (3.2, 161)	0.307
CEA (ng/mL)	2.37 (1.34, 3.60)	2.34 (1.72, 3.36)	0.203
CA19-9 (U/mL)	25 (13.67, 39.5)	16 (3.51, 29.38)	0.830
PT (s)	12.4 ± 1.52	11.8 ± 1.07	0.640
INR	1.13 ± 0.14	1.06 ± 0.10	0.041
TT (s)	18.5 ± 1.33	18.4 ± 1.11	0.756
Fib (g/L)	3.20 ± 1.44	2.77 ± 1.15	0.245
RBC (10^12^/L)	4.37 ± 0.94	4.39 ± 0.84	0.936
HGB (g/L)	131 ± 24.7	136 ± 26	0.505
HCT (L/L)	0.40 ± 0.07	0.42 ± 0.07	0.490
MCV (fL)	93.32 ± 9.19	95.45 ± 8.16	0.381
MCH (pg)	30.34 ± 3.18	31.14 ± 3.48	0.393
MCHC(g/dL)	325 ± 9.9	325.59 ± 14.84	0.867
RDW (fL)	51.26 ± 8.53	49.89 ± 7.34	0.537
PLT (10^9^/L)	116 ± 71.1	128 ± 100.9	0.638
WBC (10^9^/L)	4.69 ± 2.27	5.39 ± 4.37	0.469
Neutrophils (%)	62.91 ± 11.15	58.87 ± 13.85	0.250
Lymphocytes (%)	25.58 ± 10.97	28.96 ± 12.22	0.298
Monocytes (%)	8.4 (7.4, 9.4)	8.4(7.6, 9.6)	0.573
Eosinophils (%)	2.1 (1.2, 4.2)	2 (1.5, 3.45)	0.859
Basophils (%)	0.5 (0.3, 0.7)	0.5 (0.4, 0.7)	0.350
TB (umol/L)	20.5 ± 11.16	16.60 ± 6.77	0.140
DB (umol/L)	7.94 ± 5.72	5.89 ± 2.63	0.110
IB (umol/L)	12.56 ± 7.23	10.72 ± 4.72	0.287
ALT (IU/L)	27 (21, 37)	29 (23, 40)	0.982
AST (IU/L)	38 (33, 51)	34 (23, 53)	0.750
AST/ALT	1.46 (1.16, 1.75)	1.2 (0.96, 1.68)	0.061
ALP (IU/L)	163 ± 79.8	140 ± 117.9	0.421
GGT (IU/L)	76 (50, 182)	90 (51, 187)	0.808
CK (IU/L)	56 (38, 86)	65 (41.5, 99)	0.317
LDH (IU/L)	200 (172, 289)	182 (152, 215)	0.274
HBDH (IU/L)	145 (122, 222)	137 (111, 167)	0.292
TBA (µmol/L)	14.2 (7.5, 19.9)	9.1 (3.6, 2.2)	0.157
TP (g/L)	69.55 ± 5.21	69.52 ± 6.40	0.986
Albumin (g/L)	37.98 ± 4.43	40.36 ± 4.63	0.064
Globulin (g/L)	31.54 ± 6.18	29.18 ± 5.86	0.166
ALB/GLB	1.25 ± 0.29	1.46 ± 0.41	0.046
Glucose (mmol/L)	5.19 ± 1.16	4.97 ± 0.47	0.372
UREA (mmol/L)	4.81 ± 1.71	4.73 ± 1.77	0.866
Creatinine (umol/L)	74.68 ± 26.47	75.59 ± 19.53	0.889
eGFR (mL/min/1.73 m²)	91.11 ± 22.43	91.08 ± 19.77	0.996
Cystatin C (mg/L)	1.11 ± 0.39	1.01 ± 0.34	0.626
UA (umol/L)	322.92 ± 108.08	342.78 ± 109.92	0.515
TC (mmol/L)	3.70 ± 0.95	3.83 ± 0.81	0.576
HDL-C (mmol/L)	1.06 ± 0.29	1.05 ± 0.32	0.886
LDL-C (mmol/L)	2.24 ± 0.72	2.24 ± 0.63	0.994

Note: BMI: body mass index; PIVKA: protein induced by vitamin k absence or antagonist-II; AFP: alpha-fetoprotein; CEA: carcinoembryonic antigen; CA19-9: cancer antigen 19-9; PT: prothrombin time; INR: international normalized ratio; TT: thrombin time; Fib: Fibrinogen; RBC: red blood cell count; HGB: hemoglobin; HCT: hematocrit; MCV: mean corpuscular volume; MCH: mean corpuscular hemoglobin; MCHC: mean corpuscular hemoglobin concentration; RDW: red cell distribution width; PLT: platelet count; WBC: white blood cell count; BASO: basophils percentage; TB: total bilirubin; DB: direct bilirubin; IB: indirect bilirubin; ALT: alanine aminotransferase; AST: aspartate aminotransferase; ALP: alkaline phosphatase; GGT: gamma-glutamyl transferase; CK: creatine kinase; LDH: lactate dehydrogenase; HBDH: hydroxybutyrate dehydrogenase; TBA: total bile acids; TP: total protein; eGFR: Estimated Glomerular Filtration Rate; UA: uric acid; TC: total cholesterol; HDL-C: High-Density Lipoprotein Cholesterol; LDL-C: low-density lipoprotein cholesterol.

**Figure 1:**
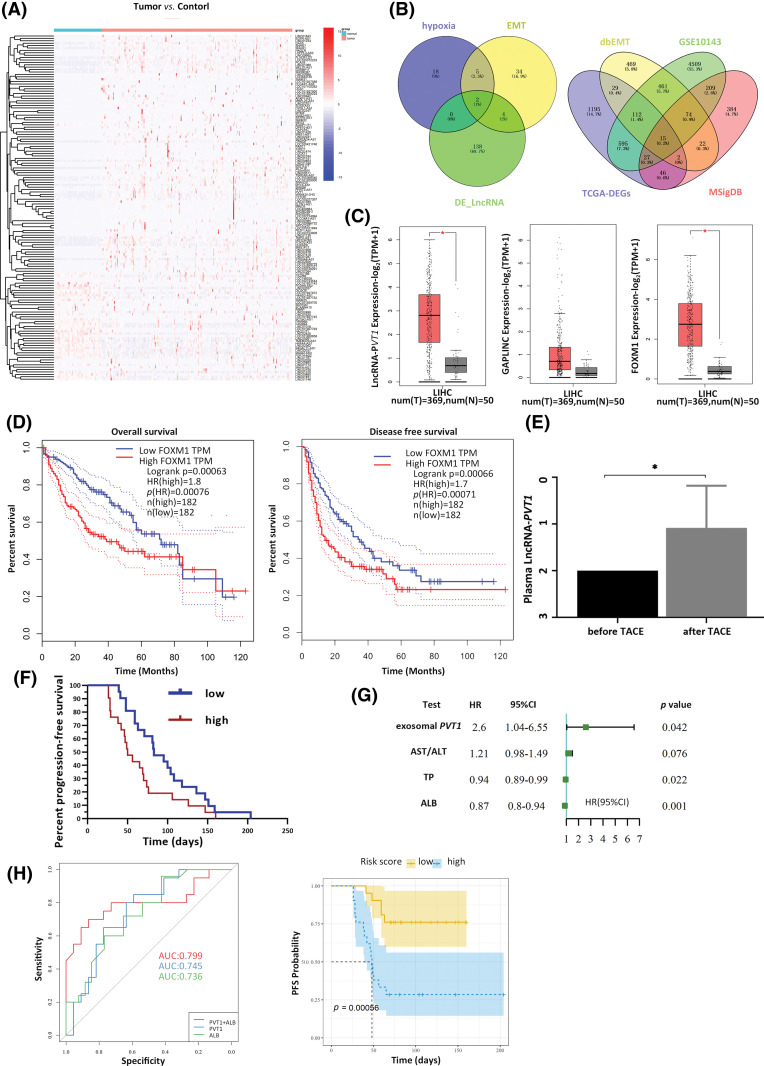
Expression of plasma exosomal lncRNA-PVT1 and its effect on post-transarterial chemoembolization (TACE) progression-free survival (PFS). (A) Differentially expressed lncRNAs were identified within the TCGA LIHC dataset. Among these differentially expressed lncRNAs, two were found to be associated with hypoxia and the epithelial-mesenchymal transition (EMT). (B) Of the differentially expressed mRNAs, 15 were related to hypoxia and EMT. (C) Compared to that in para-tumor tissue, the expression of lncRNA-*PVT1* was significantly upregulated in Hepatocellular Carcinoma tissue. Increased expression of GAPLINC in Hepatocellular Carcinoma tissue, compared to that in para-tumor tissue, which did not exhibit statistical significance. FoxM1 expression was significantly upregulated in Hepatocellular Carcinoma tissue compared to that in para-tumor tissue. (D) FoxM1 expression affected the overall survival time and disease-free survival period. (E) The relative expression of plasma exosomal lncRNA-*PVT1* increased after TACE. (F) Survival analysis of PFS based on changes in the relative expression of plasma exosomal lncRNA-*PVT1* post-TACE. (G) multivariate risk model combining plasma exosomal lncRNA-*PVT1* and Albumin to evaluate the PFS. (H) The AUC of the model was 0.799. *p < 0.05, AST: aspartate aminotransferase, ALT: alanine aminotransferase, TP: total protein, ALB: albumin.

The Liver Hepatocellular Carcinoma dataset from TCGA was used to identify differentially expressed lncRNAs between tumor and para-cancerous tissues, yielding 144 differentially expressed lncRNAs (Fig. 1A). Among these, 25 were associated with hypoxia, whereas 45 were associated with EMT. Based on the Venn diagram intersection, two lncRNAs, lncR-*PVT1* and GAPLINC, possibly implicated in HCC-associated hypoxia and EMT were identified. In parallel, combining expression profile data from TCGA, dbEMT, and MSigDB databases, followed by the Venn diagram intersection, 15 mRNAs potentially linked to HCC-related hypoxia and EMT were revealed (Fig. 1B, Table S9). A comprehensive literature review was performed to match lncR-*PVT1* and GAPLINC with 15 candidate mRNAs, which aided in the determination of target molecules. Compared to that in para-carcinoma tissues, both lncRNA-*PVT1* and GAPLINC showed elevated expression in carcinoma tissues. Notably, the difference in lncRNA-*PVT1* expression was significant. The expression level of FoxM1 significantly increased relative to that in para-carcinoma tissues (Fig. 1C). Based on median relative expression levels, the subjects were divided into high- and low-FoxM1-expression groups, each comprising 182 cases. The overall and disease-free survival times of the high-FoxM1-expression group were significantly shorter than those of the low-expression group. The effect on disease-free survival was most pronounced within the first year, as the high-expression group experienced a 60% overall decline in disease-free survival within 20 months. High FoxM1 expression corresponded to lower survival rates within 80 months compared to those in the low-expression group for the total survival time (Fig. 1D).

### Plasma exosomal lncRNA- *PVT1* can be used to assess residual cancer invasiveness post-TACE

Initially, 85 participants were enrolled. Six participants were excluded after applying the exclusion criteria, which included incomplete medical histories, poor general health precluding surgery, and distant metastases, six participants were excluded, leaving 79 patients for subsequent analyses. To ensure uniformity and to mitigate potential confounding effects, all participants underwent the same TACE procedure, employing drug-eluting microspheres combined with epirubicin for chemoembolization of the tumor-feeding arteries. Exosomes were extracted from the circulation to assess the potential use of exosomal biomarkers for recurrence prediction for further investigation. The identification of plasma exosomes is shown in Fig. S1.

Post-TACE, the relative expression of exosomal lncRNA-*PVT1* in the plasma was significantly elevated, 1.92-fold compared to pre-surgery levels (*p* = 0.046, Fig. 1E). A subsequent survival analysis revealed that patients with exosomal lncRNA-*PVT1* expression above the median level were categorized as high risk, whereas those below the median level constituted low-risk group. The PFS was significantly shorter in the high-risk group than those in the low-risk group. The median PFS for the low-risk group was 83 days, whereas it was 50 days for the high-risk group (*p* < 0.05, Fig. 1F). Patients with a PFS below the median were classified as having early recurrence, whereas those with PFS above the median (including patients without recurrence) were considered to have late recurrence. Basic patient details and pre-TACE laboratory test results were compared between the two groups. After excluding data from lost follow-ups or those that did not comply with the prescribed visit schedules, the final cohort included 52 patients. The general patient condition and preoperative laboratory results showed no significant differences in the median PFS between the early and late recurrence groups (Table 1).

Compared to those pre-TACE, the red blood cell (RBC) count, hemoglobin (HGB), mean corpuscular volume (MCV), and mean corpuscular hemoglobin concentration (MCHC) were significantly reduced after the procedure (*p* < 0.05). WBC and neutrophils increased postoperatively, whereas eosinophils, lymphocytes, and basophils significantly decreased (*p* < 0.05). Bilirubin, total bile acid, aminotransferase, serum protein, LDH, and HBDH levels significantly increased postoperatively compared to preoperative measurements (*p* < 0.05). The effects on kidney function were mixed. Urea, cystatin c, and uric acid decreased significantly (*p* < 0.05), whereas the changes in creatinine and estimated glomerular filtration rate (eGFR) postoperatively were not statistically significant (*p* > 0.05). Fasting blood sugar levels increased significantly after surgery (*p* < 0.001). In contrast, cholesterol levels, including total cholesterol, high-density lipoprotein cholesterol, and low-density lipoprotein cholesterol, were significantly lower than preoperative levels (*p* < 0.001, Table S10).

Univariate Cox regression analysis was used to screen for prognostic indicators that could affect PFS (*p* < 0.1 as the screening criterion). The relative postoperative plasma exosomal LncRNA-*PVT1* expression level and AST/ALT levels were risk factors for the postoperative PFS. In contrast, serum proteins were protective factors against postoperative PFS (Fig. 1G). The following prognostic model was established using multivariate Cox regression analysis: exosome PVT × 1.05 + ALB × (−0.16). Based on the median model score, patients were classified into high-risk (scores above the median) or low-risk (scores below the median) groups. Survival analysis showed that PFS in the high-risk group was approximately 50 days shorter than that in the low-risk group, with a significant difference (*p* < 0.001). The performance of the multivariate Cox regression model was evaluated using ROC curves. The areas under the curve (AUCs) for plasma exosomal lncRNA-*PVT1* expression and albumin levels, when used separately, were 0.745 and 0.736, respectively. When combined, the AUC increased to 0.799, with a cutoff value of −5.267. The corresponding sensitivity and specificity were 0.864 and 0.7, respectively (Fig. 1H).

### Exosome-driven invasion in hypoxia-induced HCC

HepG2, Hep3B, and Huh7 cells were cultured separately to compare their invasive, migratory, and proliferative abilities (Fig. S2a–c). Findings suggested that among the HCC cell lines, HepG2 cells have the strongest invasive ability and should be considered as donor cells, whereas Huh7 cells, with the weakest invasive capacity, should serve as recipient cells. After induction with 200 µmol/L of CoCl_2_ for 24 h to induce hypoxia, HepG2 cell morphology was observed and photographed under a microscope. Following hypoxia treatment, the HCC cells displayed signs of apoptosis and morphologically transitioned from quasi-round to fusiform shapes (Fig. S2d,e). HIF-1α expression was upregulated, showing a statistically significant difference compared to that before the treatment (*p* = 0.009, Fig. S2f).

**Figure 2:**
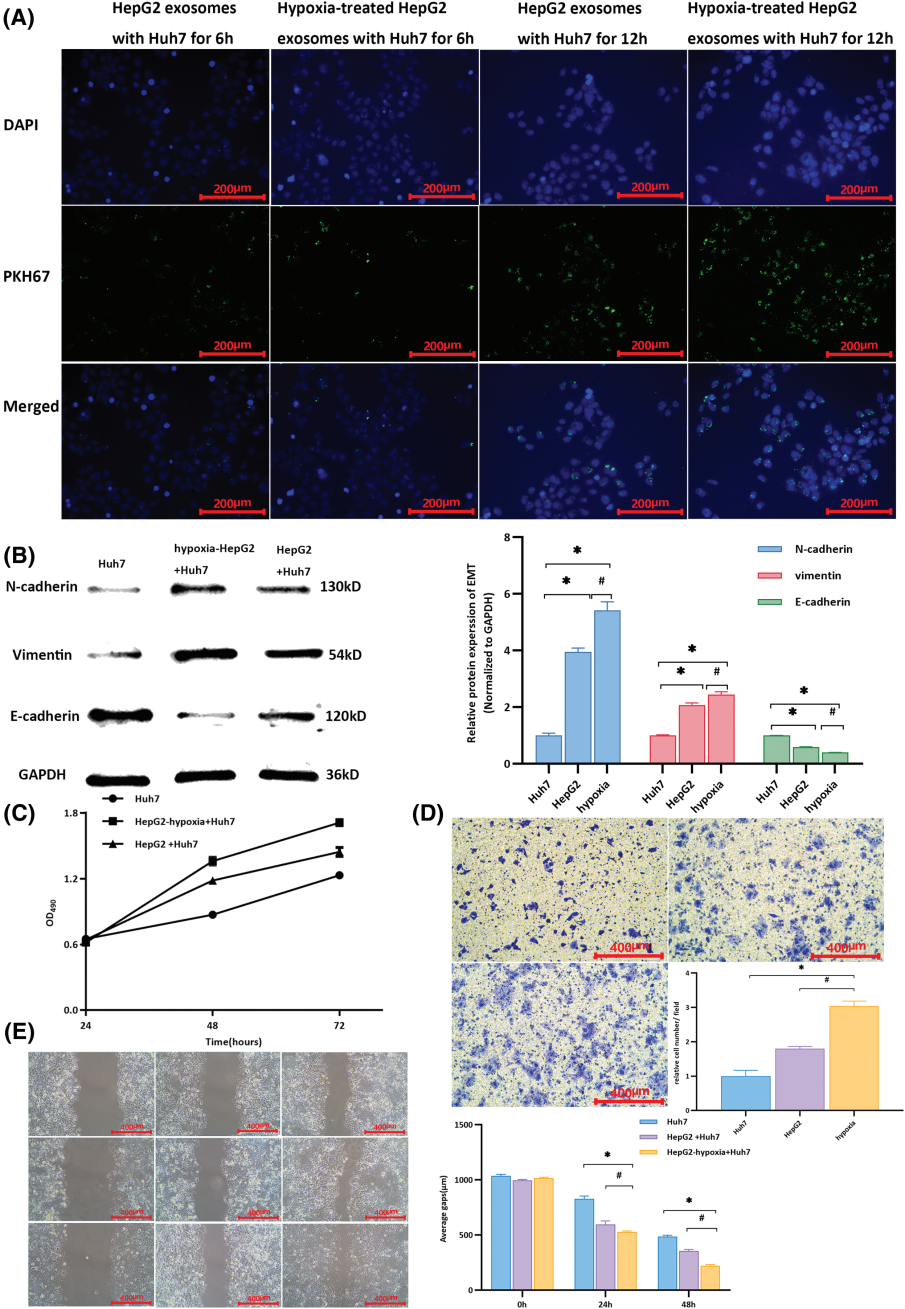
Changes in Huh7 cells after co-culturing with exosomes from different cells. (A) Cells were cultured for 6 h, and the uptake of exosomes by recipient Huh7 cells was observed through fluorescence microscopy. Upon co-cultivation for up to 12 h, there was an increase in the intensity of green fluorescence compared to that at 6 h, indicating more exosome uptake by Huh7 cells. Moreover, a significant aggregation phenomenon was observed. (B) Compared with that in untreated Huh7 cells, the expression of E-cadherin was downregulated, whereas that of N-cadherin and vimentin was upregulated in Huh7 cells after co-culturing them with exosomes from HepG2 and hypoxia-induced HepG2 cells. * indicates a comparison with untreated Huh7 cells (*p* < 0.05); # indicates a comparison with the HepG2 group (*p* < 0.05). (C) The proliferative activity of Huh7 cells co-cultured with exosomes from hypoxia-induced HepG2 cells was higher than that of Huh7 cells co-cultured with exosomes from untreated HepG2 cells and untreated Huh7 cells. (D) Using untreated Huh7 cells as controls, after co-culture with exosomes from hypoxia-induced HepG2 cells, the relative number of Huh7 cells per field in the lower chamber was greater than that in Huh7 cells co-cultured with HepG2 exosomes. (E) Compared with the scratch distance of untreated Huh7 cells, the average scratch distance of Huh7 cells after co-culture with exosomes from hypoxia-induced HepG2 cells was shortest at 24 and 48 h, followed by that of Huh7 cells co-cultured with HepG2 exosomes. *Compared with untreated Huh7 cells, *p* < 0.05; #compared with HepG2+Huh7, *p* < 0.05.

Exosomes were enriched in the HepG2 cell culture medium. Electron microscopy, western blotting, and particle size analysis revealed the characteristics of the exosomes (Fig. S2g–j). After hypoxia induction, exosomal lncRNA-PVT1 extracted from the culture medium was evaluated in HepG2 cells (Fig. S2k). Exosomes were enriched in the culture medium of HepG2 cells after hypoxia induction and co-cultured with recipient Huh7 cells. After co-culturing for 6 and 12 h, green fluorescence increased in recipient Huh7 cells following exosome uptake (Fig. 2A). These results demonstrated that recipient Huh7 cells can take up exosomes. As time progresses, the number of exosomes taken up by recipient cells gradually increases.

Differences in the expression of EMT-related markers in Huh7 cells were observed after the uptake of different extracellular vesicles. After co-culture with exosomes from HepG2 HCC cells subjected to hypoxia (HepG2-hypoxia), E-cadherin expression in Huh7 cells was downregulated compared to that in untreated Huh7 cells, whereas N-cadherin and vimentin expression was significantly upregulated (*p* < 0.001). After co-culture with exosomes from highly invasive HepG2 cells subjected to hypoxia (HepG2-hypoxia), the expression of E-cadherin in Huh7 cells was downregulated compared to that in Huh7 cells co-cultured with exosomes from HepG2 cells without hypoxia induction; however, expressions of N-cadherin and vimentin were elevated (*p* = 0.008, *p* = 0.012, *p* < 0.001, respectively, Fig. 2B). These findings suggest that after co-culturing exosomes from highly invasive HCC cells with minimally invasive recipient cells, the EMT capability of the recipient cells was promoted. Moreover, this effect was stronger when recipient cells were co-cultured with exosomes from highly invasive HCC cells subjected to hypoxia. The proliferative activity of Huh7 cells peaked at 72 h and was highest when cells were co-cultured with exosomes from highly invasive liver cancer cells subjected to hypoxia (HepG2-hypoxia, Fig. 2C). In the Transwell chamber, the highest relative cell count per field of view was observed in the lower chambers (Fig. 2D). The average scratch distance was shortest at both 24 and 48 h (Fig. 2E). Exosomes secreted by highly invasive liver cancer cells (HepG2) under hypoxia could be taken up by recipient Huh7 cells, resulting in upregulation of the expression of EMT-related markers and the promotion of proliferation, invasion, and migration in Huh7 cells.

**Figure 3:**
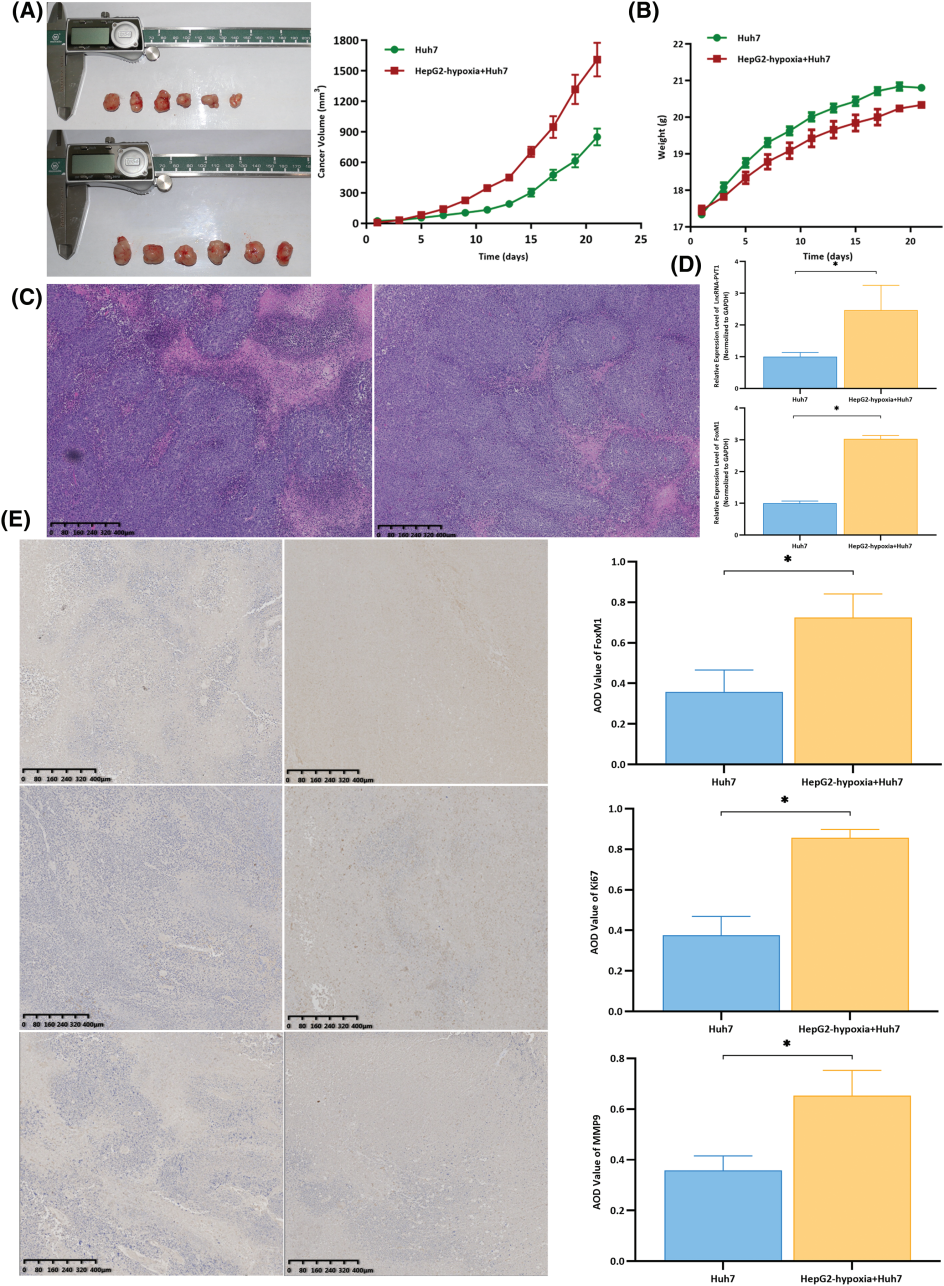
Subcutaneous xenografts using differentially treated Huh7 cells were generated using nude mice to observe tumor formation and compare Hepatocellular Carcinoma (HCC) invasiveness. (A) Compared with those in the group treated with Huh7 cells, the tumor volumes in nude mice inoculated with Huh7 cells co-cultured with exosomes from hypoxia-induced HepG2 cells were larger, and tumors grew faster. (B) At the time of euthanasia, the untreated group of nude mice weighed the most, with an average weight increase of approximately 4 g. The nude mice, for which the weight increased by an average of approximately 3 g, were inoculated with Huh7 cells co-cultured with exosomes from hypoxia-induced HepG2 cells. (C) The hematoxylin and eosin staining results revealed that the tumor tissues were mostly distributed in clustered foci. The tissue structure was still acceptable, with tumor cells showing significant atypia, an uneven size, and an irregular shape. The nuclear division could be observed in some cells. (D) Compared with that in the control group, the expression of lncRNA-*PVT1* and FoxM1 was upregulated in the tumor tissues of nude mice inoculated with Huh7 cells co-cultured with exosomes from hypoxia-induced HepG2 cells. The differences were statistically significant (*p* = 0.018, *p* < 0.001). (E) Compared with that in the control group, FOXM1, Ki67, and MMP9 expression was upregulated in the tumor tissues of nude mice inoculated with Huh7 cells co-cultured with exosomes from hypoxia-induced HepG2 cells. The differences were statistically significant (*p* = 0.005, *p* < 0.001, *p* = 0.003). *Compared with untreated Huh7 cells, *p* < 0.05.

Untreated Huh7 cells were used as controls. These were compared to Huh7 cells co-cultured with exosomes from hypoxia-induced HepG2 cells, which were subcutaneously inoculated into nude mice to observe tumor formation, thereby assessing the effect of exosomes on the invasiveness of liver cancer. The volumes of tumors formed in mice inoculated with Huh7 cells co-cultured with exosomes from hypoxia-induced HepG2 cells were larger than those in the control group (Fig. 3A), yet they had a lighter body weight. (Fig. 3B). Hematoxylin and eosin staining of tumor tissues revealed a predominantly nodular distribution with a relatively normal tissue structure. The tumor cells exhibited notable heterogeneity with an uneven size, irregular shape, and visible nuclear division (Fig. 3C). Compared with that in the control group, the expression of lncRNA-*PVT1* and FoxM1 was significantly upregulated in the tumor tissues of mice inoculated with Huh7 cells co-cultured with exosomes from hypoxia-induced HepG2 cells (*p* = 0.018 and *p* < 0.001, respectively). Additionally, the expression of miR-345-5p was downregulated, with significant differences between the groups (*p* = 0.035, Fig. 3D). Immunohistochemical staining revealed that the expression of FOXM1, Ki67, and MMP9 in the tumor tissues of mice inoculated with Huh7 cells co-cultured with exosomes from hypoxia-induced HepG2 cells significantly increased (*p* = 0.005, *p* < 0.001, *p* = 0.003, Fig. 3E) when compared to that in the control group.

### The competitive inhibition of miR-354-3p mediated by lncRNA- *PVT1* facilitates EMT in HCC via *FOXM1* regulation

A comparison of the effects of the overexpression plasmid lncRNA-*PVT1* and siRNA expression showed significant differences. The expression of lncRNA-*PVT1* with PCDNA3.1-lncRNA-*PVT1* was significantly upregulated when compared to that of lncRNA-*PVT1* with PCDNA3.1-NC (*p* = 0.004). In contrast, the expression of lncRNA *PVT1* was significantly downregulated when compared to that observed with si-NC (*p* = 0.006, Fig. S3a).

**Figure 4:**
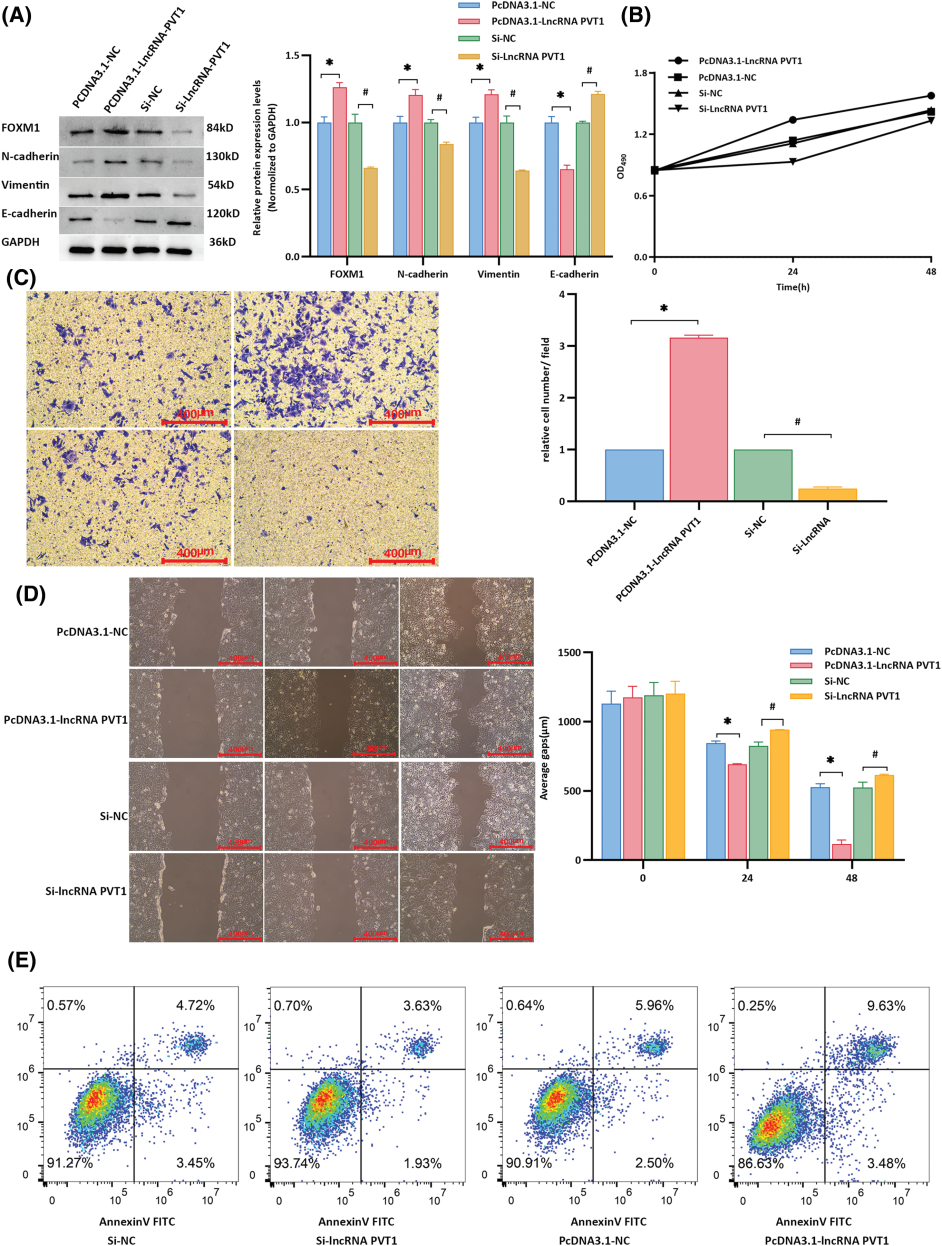
Influence of lncRNA-*PVT1* overexpression and knockdown on epithelial-mesenchymal transition in Huh7 cells. (A) Following the overexpression of lncRNA-*PVT1*, compared to that in the control group, E-cadherin expression was significantly downregulated, and N-cadherin, vimentin, and FoxM1 expression was significantly upregulated (all *p* < 0.001). Conversely, silencing lncRNA-*PVT1* led to a significant decrease in the expression of N-cadherin, vimentin, and FoxM1, whereas E-cadherin expression was increased, with all differences being statistically significant (all *p* < 0.001). *Compared with PCDNA3.1-NC, *p* < 0.05; #Compared with si-NC, *p* < 0.05. (B) The proliferative activity was highest at 24 and 48 h following the overexpression of lncRNA-*PVT1*. In contrast, the knockdown of lncRNA-*PVT1* resulted in the lowest proliferative activity at 24 and 48 h. (C) The invasive capability was strongest at 24 and 48 h following the overexpression of lncRNA-*PVT1*, with the highest average number of cells per field observed. Conversely, silencing lncRNA-PVT1 reduced invasive capacity, with the lowest average number of cells per field. (D) Overexpression of lncRNA-*PVT1* enhanced cellular migration, resulting in the shortest average scratch distance after 48 h. However, upon silencing lncRNA-*PVT1*, cell migration was impaired, resulting in an average scratch distance greater than that in both the lncRNA-*PVT1* overexpression group and the blank control group. (E) Compared to that in the control group, the PCDNA3.1-lncRNA-*PVT1* group showed an increased proportion of normal cells and a decreased proportion of apoptotic and dead cells. In contrast, the si-lncRNA-*PVT1* group exhibited a reduced percentage of normal cells and an increase in apoptotic and dead cells compared to those in the control group. *Compared to NC mimics, *p* < 0.05; #Compared to Si-NC, *p* < 0.05.

After transfection with the lncRNA-*PVT1* overexpression plasmid and siRNA, the expression of EMT-related markers and the downstream FOXM1 protein was examined in Huh7 cells. Compared to that in the control, lncRNA-*PVT1* overexpression resulted in the downregulation of E-cadherin and upregulation of N-cadherin, vimentin, and FoxM1 expression. The findings showed significant differences (*p* < 0.001). In contrast, silencing lncRNA-*PVT1* led to the downregulation of N-cadherin, vimentin, and FoxM1 expression and the upregulation of E-cadherin expression compared to levels with the si-NC control (*p* < 0.001). This indicates that lncRNA-*PVT1* overexpression can upregulate the expression of the downstream FOXM1 protein, promoting EMT. In contrast, the downregulation of lncRNA-*PVT1* expression can suppress the expression of the downstream FOXM1 protein, inhibiting the acquisition of EMT-related characteristics in cells. (Fig. 4A).

MTT assay results showed that the proliferative activity of Huh7 cells was highest at 24 and 48 h when lncRNA-*PVT1* was overexpressed, whereas the proliferative activity was lowest at these time points when lncRNA-*PVT1* was silenced (Fig. 4B). Transwell assay results revealed that cells overexpressing lncRNA-*PVT1* had the strongest invasive ability at 24 and 48 h, with the highest average cell number per field of view. In contrast, cells with silenced lncRNA-*PVT1* demonstrated reduced invasive capacity, with the lowest average cell count per field of view (Fig. 4C). Scratch assay results indicated that lncRNA-*PVT1* overexpression enhanced cell migration, with the shortest average scratch distance observed after 48 h. However, the downregulation of lncRNA-PVT1 expression resulted in reduced cell migration, with an average scratch distance greater than that in the lncRNA-*PVT1* overexpression control group (Fig. 4D).

In the PCDNA3.1-NC group, 91.27% of the cells were healthy, whereas apoptosis and death occurred in 8.17% of cells. By comparison, in the PCDNA3.1-lncRNA-*PVT1* group, in which lncRNA-*PVT1* was overexpressed, an increased percentage of healthy cells (93.74%), as well as a decreased percentage of apoptotic and dead cells (5.56%) was observed. In the si-NC group, 90.91% of cells were healthy, and 8.46% of cells underwent apoptosis or death. In comparison, the si-lncRNA-*PVT1* group, with decreased lncRNA-*PVT1* expression, showed a decrease in the proportion of healthy cells (86.63%) and an increase in the ratio of apoptotic to dead cells (13.11%). These results suggest that lncRNA-*PVT1* inhibits apoptosis (Fig. 4E).

The online databases Starbase v3.0 and Lncbase v2.0 were used to show that lncRNA-*PVT1* regulates *FoxM1* via the ceRNA mechanism through miR-345-5p (Fig. S3b). miR-345-5p expression was lowest in highly invasive HepG2 cells and highest in less invasive Huh7 cells (Fig. S3c). The dual-luciferase reporter gene assay results indicated that miR-345-5p directly targets lncRNA-*PVT1*-5:1. However, this targeting effect was not observed when the seed sequence was mutated. Similarly, miR-345-5p could directly target the 3′-UTR regions of *FoxM1*; however, this effect was also mitigated after a seed sequence mutation. These findings suggest that miR-345-5p can competitively bind lncRNA-*PVT1* and *FoxM1*, exerting an inhibitory effect (Fig. S3d,e). Based on the treatment of HCC Huh7 cells with miR-345 mimics and miR-345 ASO, the overexpression of miR-345-5p inhibited the proliferation, invasion, and migration of Huh7 cells and promoted apoptosis, whereas the suppression of miR-345-5p expression increased the proliferation, invasion, and migration of Huh7 cells, while inhibiting cell death (Fig. S4a–e).

### Exosomal lncRNA *-PVT1* promotes EMT in liver cancer

**Figure 5:**
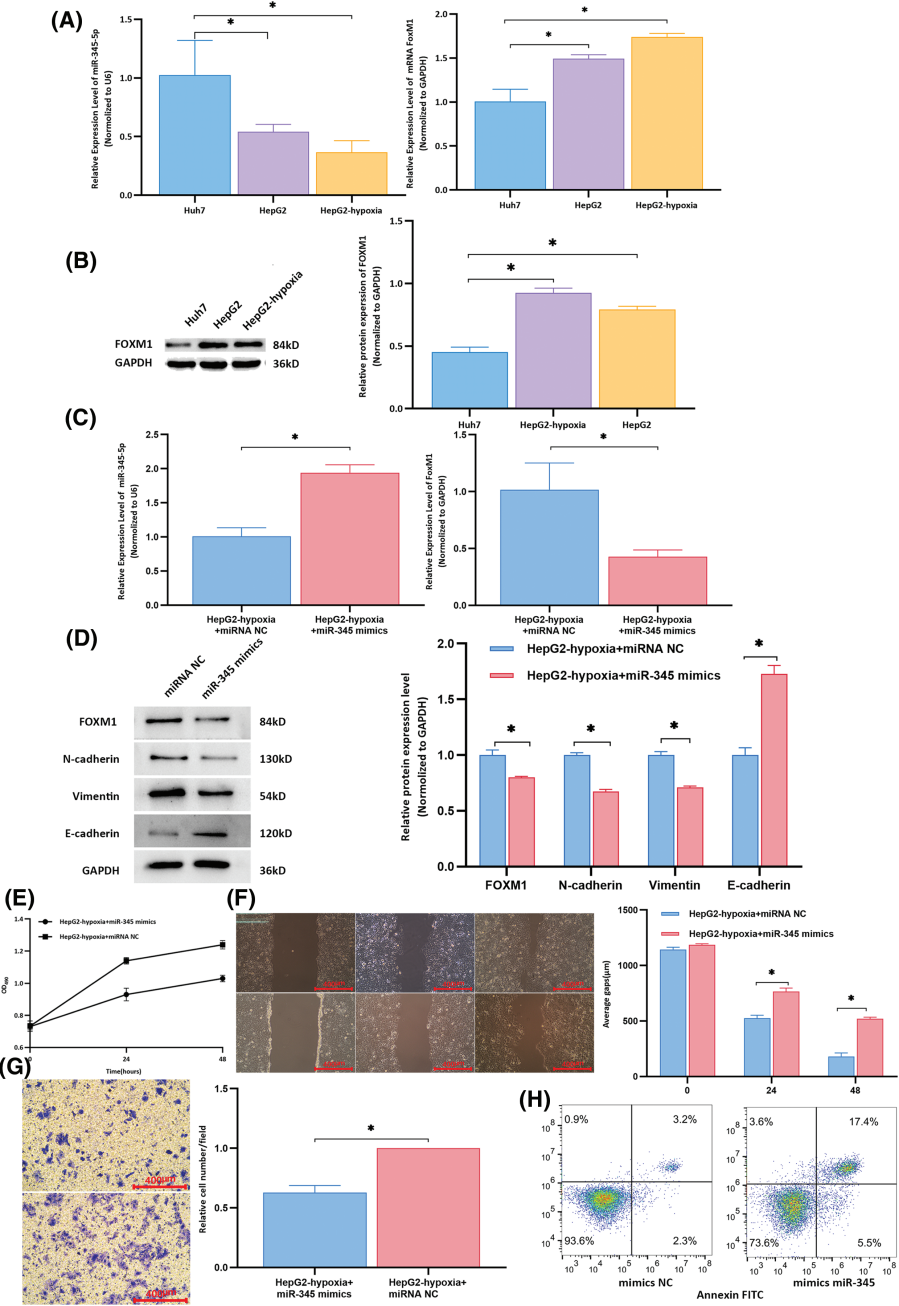
Rescue experiments to determine the expression of target molecules and biological functions in recipient cells. (A) Compared to that in the control group, the expression of miR-345-5p in Huh7 cells co-cultured with HepG2 cell-derived exosomes was downregulated (all *p* < 0.05). The mRNA level of *FoxM1* in the control group was lower than that in the group of Huh7 cells co-cultured with exosomes from HepG2 cells (all *p* < 0.001). (B) The protein expression of FOXM1 in the control group was lower than that in the group of Huh7 cells co-cultured with exosomes from HepG2 cells (all *p* < 0.001). (C) Compared to that in the control group, the expression of miR-345-5p in the experimental group was significantly upregulated (*p* < 0.001), and the mRNA level of *FoxM1* was significantly downregulated (*p* = 0.014). (D) Compared to those in the control group, the experimental group exhibited downregulation of the protein expression levels of FOXM1, N-cadherin, and vimentin, whereas the relative expression of E-cadherin was increased in the experimental group. (E) At 24 and 48 h, the proliferative activity of control group cells was higher than that of the experimental group. (F) Wound healing assay results indicated that compared to that in the control group, the average migration distance of cells at 24 and 48 h was decreased in the experimental group. (G) Transwell assay results showed that compared to that in the control group, the average number of cells per field in the lower chamber was reduced in the experimental group. H. In the experimental group, normal cells accounted for 73.6% of cells, whereas apoptotic and dead cells comprised 22.9%. In the control group, normal cells constituted 93.6% of cells, with apoptotic and dead cells representing 5.5%. *Compared to NC mimics or control, *p* < 0.05.

Compared to that in the control group, the expression of miR-345-5p was downregulated in Huh7 cells co-cultured with HepG2 cell-derived exosomes (*p* = 0.039) and in those co-cultured with exosomes from hypoxia-induced HepG2 cells (*p* = 0.008, Fig. 5A). Additionally, both the mRNA and protein levels of FoxM1 were lower in the control group than in Huh7 cells co-cultured with HepG2 cell exosomes (*p* < 0.001; Fig. 5B). Exosomes from hypoxia-treated HepG2 cells + miR-345 mimics were used as the experimental group, and exosomes from hypoxia-treated HepG2 cells + miRNA NC were used as the control group and co-cultured with recipient Huh7 cells. miR-345 overexpression inhibited the effects of exosomes from highly invasive cells on recipient cells, reducing the proliferation, invasion, and migration of recipient cells, promoting apoptosis in recipient cells, and decreasing the inhibitory effect of exosomes from highly invasive cells on the apoptosis of recipient cells (Fig. 5C–H).

## Discussion

TACE is a localized treatment for liver cancer that has demonstrated good therapeutic efficacy in improving the quality of life and overall survival time of patients [15]. It is now a common treatment strategy [16]. Studies have aimed to develop prognostic prediction models to enhance postoperative therapeutic assessments and aid in clinical decision-making [17]. In this study, despite a decrease in RBC and HGB post-TACE, no clinical significance was found, because the mean values remained within the reference range. However, vigilance is needed for patients with anemia because of the potential for a further reduction in red blood cell levels postoperatively. Moreover, changes in leukocyte counts were also observed, suggesting that TACE might alter the tumor-derived microenvironment, thereby affecting immune cell infiltration. Based on pre-TACE laboratory results, a systemic inflammation model constructed by Xiao et al. indicated that neutrophil, albumin, and lymphocyte count achieved a prognostic predictive value of approximately 65% [18]. A multicenter study incorporating tumor size, the number of tumors, and AFP levels in the model achieved a prognostic predictive value of approximately 70% [19]. In this study, a combination predictive model based on plasma exosomal lncRNA-*PVT1* has enhanced the effectiveness of existing assessment schemes and could potentially serve as a new target for postoperative therapeutic evaluations.

Further experiments are needed to explore the role of exosomes in mediating intercellular communication among liver cancer cells and in regulating microenvironments. Our experiments confirmed that after co-cultivation with exosomes from highly invasive cells, poorly invasive cells show enhanced proliferation, invasion, and migration, with an upregulated expression being observed for EMT-related markers; this result is like previous findings [20]. Some researchers have discovered that the highly invasive liver cancer cell line MHCC97H can interact with a poorly invasive liver cancer cell line (MHCC97L) and alter its proliferation, invasion, and migration capabilities. Exosomes from highly invasive liver cancer cell lines downregulate E-cadherin expression and upregulate vimentin expression in recipient cells. Mechanistic verification indicated that after treatment with exosomes from highly invasive liver cancer cells, biological changes in recipient cells were induced by MAPK/ERK signaling, thereby facilitating EMT [21]. *In vivo* experiments have shown that exosomes from highly invasive liver cancer cells promote intrahepatic liver cancer recurrence in nude mice [22]. Additional research on exosomal contents based on the differential expression of proteins within exosomes in an HCC cell-conditioned medium showed that some specific proteins were highly enriched in exosomes secreted by Slug-overexpressing liver cancer cells. These proteins participate, fully or in part, in EMT induction [23]. MiRNA sequencing of exosomes from highly invasive liver cancer cells revealed many differentially expressed miRNAs; among them, miR-92a-3p was the most abundant. Moreover, exosomal miR-92a-3p levels in the plasma of mice heterogeneously transplanted with highly metastatic HCC increased. Mechanistic studies revealed that exosomal miR-92a-3p promotes EMT in recipient cancer cells by targeting PTEN and regulating its downstream Akt/Snail signaling pathway [24]. Clinical data have also indicated that increased plasma exosomal miR-92a-3p levels are associated with shorter overall and disease-free survival periods, indicative of poor prognoses for patients with liver cancer [24]. In addition, exosomes from tumor-associated fibroblasts (CAFs) can transfer miR-320a to HCC cells, thereby suppressing EMT. The loss of antitumor miR-320a in CAF exosomes can induce EMT and promote tumor progression [25]. This suggests that exosome-mediated liver cancer invasion and metastasis play crucial roles in both local and distant microenvironments. Our research utilized databases to screen lncRNAs related to hypoxia and EMT in liver tissues associated with plasma exosomes and validated their prognostic potential following TACE surgery. Therefore, this study focused on the functions of exosomal lncRNAs.

In digestive system tumors, lncRNA-*PVT1* is involved in tumor proliferation, differentiation, and EMT, thereby promoting liver cancer growth, survival, invasion, metastasis, and drug resistance [26]. In this study, lncRNA-*PVT1* overexpression increased recipient cell proliferation, invasion, and migration and suppressed apoptosis, whereas lncRNA-*PVT1* silencing reduced cell proliferation, invasion, and migration and increased apoptosis. These findings are similar to the results of Kong et al. based on gastric cancer, in which microarray technology was used to identify dysregulated lncRNAs in gastric cancer tissues. Among them, lncRNA-*PVT1* expression was abnormally upregulated in malignant gastric cancer tissues and correlated with poor patient prognosis. Both *in vivo* and *in vitro* experiments on gastric cancer cells revealed that lncRNA-*PVT1* can enhance tumor cell proliferation and invasion [27].

Determining the upstream and downstream targets of lncRNA-*PVT1* will help to clarify its key role in tumor progression. In liver cancer, lncRNA-*PVT1* can promote migration of the HCC cell line (HepG2) by regulating miRNAs [28]. LncRNA-*PVT1* can directly bind to the FoxM1 protein and upregulate its expression by increasing its stability during transcriptional regulation. Moreover, FoxM1 directly binds to the promoter of LncRNA-*PVT1*, activating its transcription. The positive feedback loop between lncRNA-*PVT1* and FoxM1 plays a crucial role in promoting tumor growth and invasion [29,30]. LncRNA-*PVT1* can promote the progression of hepatitis B-related HCC by interfering with histone methylation of the *c-Myc* promoter [31]. Furthermore, lncRNA-*PVT1* also mediates the ceRNA regulatory effect of FoxM1 on EMT in tumors. In this study, we used databases to screen miRNAs that could simultaneously bind to lncRNA-PVT1 and FoxM1 with opposite expression trends. We confirmed their expression in liver cancer cell lines and identified miR-345-5p as a target molecule for the ceRNA mechanistic study. Using a dual-luciferase reporter gene experiment, we confirmed the binding sites of miR-345-5p on lncRNA-*PVT1* and *FoxM1*. Mechanistic verification showed that the proliferation and invasion capabilities of poorly invasive liver cancer cells could be improved via miR-345 overexpression; conversely, inhibiting the miR-345 expression can promote the proliferation and invasion of these cancer cells. Rescue experiments confirmed that miR-345-5p overexpression could mitigate the enhancing effect of highly invasive liver cancer cells on the proliferation, invasion, and migration of poorly invasive liver cancer cells and restore the suppression of apoptosis.

In ovarian cancer, Yi et al. [32] found that lncRNA-*PVT1* acts as a sponge and binds to miR-370 at two binding sites, thereby promoting the proliferation, migration, and invasiveness of ovarian cancer cells. After miR-370 inhibition, FoxM1 expression could not be silenced; lncRNA-*PVT1* can directly bind to and stabilize the FoxM1 protein, both of which promote the malignant progression of ovarian cancer. Furthermore, inhibiting FoxM1 or overexpressing miR-370 can reverse the promoting effect of lncRNA-*PVT1* on the malignant progression and chemoresistance of ovarian cancer.

This study has yielded important findings; however, several limitations should be acknowledged. The prospective cohort sample size is limited, and the study was conducted at a single center, which may not fully capture the heterogeneity of all HCC patients, potentially impacting the robustness of the statistical analyses and prognostic models. Given the complexity of hypoxic conditions and the EMT process, the cell lines used in this study may not entirely replicate the physiological conditions observed *in vivo*. Although lncRNA-PVT1 has been identified as a critical factor, further research is needed to validate its specific role across different HCC subtypes and to explore its function in other cancer types. To enhance the comprehensiveness and generalizability of the findings, future studies should aim to increase the sample size, involve multicenter collaborations, and conduct longer follow-up periods. Additionally, further investigation into the underlying molecular mechanisms and pathways is essential to achieve a more complete understanding of the invasion and metastasis processes in HCC.

## Conclusions

In summary, the expression of exosomal lncRNA-PVT1 in highly invasive cells is upregulated after hypoxia induction, and its effects on poorly invasive cells might be achieved through a ceRNA mechanism mediated by miRNA-345-5p. This positively regulates downstream FoxM1 expression, enabling poorly invasive cells to acquire EMT capabilities. Therefore, plasma exosomal lncRNA-*PVT1* could serve as a potential biological marker for the prognostic evaluation of patients with liver cancer after TACE.

## Supplementary Materials

Figure S1 **The identification of plasma exosomes.** **a** Electron microscopy scans of re-suspended plasma exosomes revealed vesicles approximately 100nm in size, presenting saucer-like structures. **b** Western blot tests confirmed the presence of the exosomal membrane surface markers CD63, TSG101, and the liver-derived exosomal membrane surface marker ASGPR1—signifying positive results. **c** Employing nanoflow cytometry to track and analyze Brownian motion of each particle indicated that the average diameter of plasma exosomes in hepatocellular carcinoma patients was 68.09 nm, with a concentration of 6.10E+10 particles/mL (**d**).

Figure S2 **The proliferative activity, invasive ability, and migratory ability of different cell lines were compared, and a hypoxic microenvironment for hepatocellular carcinoma cells was simulated using CoCl2.** **a** The MTT experiment compared the proliferative activities of various groups of cells, with HepG2 exhibiting the highest activity. **b** Transwell experiments revealed that the invasiveness of HepG2 was higher than that of Hep3B, while Huh7 demonstrated the lowest invasive capability. **c** HepG2 exhibited the greatest migratory ability in the scratch test among all the cells tested. **d** HepG2 tended to grow adhered to the surface, forming a quasi-circular pattern. **e** After being treated with CoCl2, apoptotic cells appeared and HepG2 morphed into a fusiform shape. **f** The relative expression levels of HIF-1α underwent changes before and after the induction of hypoxia. **g** Under the electron microscope, tea tray-like vesicles with a diameter of approximately 100nm were observed, which allowed for the observation of the bilayer nature of exosomes. **h** Western blot (WB) results indicated that extracellular vesicles expressed exosomal membrane protein-related markers CD63, TSG101, and HSP70. **i** Particle size analysis showed that the median diameter of the extracellular vesicles was about 76.69 nm. **j** The concentration was estimated to be approximately 9.09E+10 Particles/mL. **k** Compared to untreated HepG2 cells as the control group, the relative expression level of LncRNA-*PVT1* increased after hypoxia induction in HepG2 cells, (*p*= 0.5504). *Compared to HepG2, p<0.05.

Figure S3 **The ceRNA mechanism mediated by miR-345-5p is involved in the regulatory process of LncRNA-PVT1 and FoxM1.** **a** Compared with PCDNA3.1-NC, the expression of PCDNA3.1-LncRNA-PVT1 is significantly upregulated (*p*=0.004). In contrast, compared to Si-NC, the expression of Si-LncRNA PVT1 is significantly downregulated (*p*=0.006). *Compared with the control group, p<0.05. **b** Compared with normal tissues, miR-214-3p, miR-345-5p and miR-455-3p was upregulated. **c** miR-345-5p exhibited the lowest expression level in HepG2 and the highest in Huh7. Conversely, miR-455-3p displayed the highest expression in Huh7, with comparable levels in HepG2 and Hep3B. MiR-214-3p showed the highest expression in Huh7 and the lowest in Hep3B. **d** MiR-345-5p can directly target LncRNA-PVT1-5:1 and the 3'UTR region of FoxM1. After mutating the seed sequence, this targeting effect disappears. **e** After treating Huh7 cells with miR-345 mimics, the expression of miR-345-5p increased significantly compared to the control group mimics NC (*p*=0.005). Conversely, when Huh7 cells were treated with miR-345 ASO, the expression of miR-345-5p decreased significantly compared to the control group ASO NC (*p*=0.027). *Compared to mimics NC, p<0.05; #Compared to ASO-NC, p<0.05.

Figure S4 **The influence of miR-345-5p on EMT in hepatocellular carcinoma cells.** **a** Compared to the control group ASO NC, the protein expression levels of the downstream target FOXM1 and EMT markers N-cadherin and vimentin were increased, while the expression level of E-cadherin was decreased, with all differences being statistically significant (*p*<0.001). In hepatocellular carcinoma Huh7 cells overexpressing miR-345-5p, compared to the control group mimics NC, the expression of E-cadherin was upregulated, although the difference was not statistically significant (*p*=0.053). The expressions of the downstream target FOXM1 and EMT markers N-cadherin and vimentin were downregulated, with each showing a statistically significant difference (*p*<0.001). **b** MTT showed that compared to the control group, the Huh7 cells treated with miR-345 ASO exhibited stronger proliferative activity, while those treated with miR-345 mimics showed reduced proliferation. **c** Suppressing the expression of miR-345 can increase the invasive capability of Huh7 cells, while overexpressing miR-345 can inhibit the invasive capability of Huh7 cells. **d** Compared to the control group, Huh7 cells treated with miR-345 mimics exhibited reduced migration capability, with the average scratch distance at 24h and 48h being greater than that of the control group; in contrast, Huh7 cells treated with miR-345 ASO showed increased migration capability, with the average scratch distance at both 24h and 48h being smaller than that of the control group. **e** In the mimics NC group, normal cells accounted for 90.2%, and apoptotic and dead cells constituted 8.9%; compared to the mimics NC group, the miR-345 mimics group showed a decrease in the proportion of normal cells to 64.3% and an increase in apoptotic and dead cells to 30.4%. In the ASO NC group, normal cells made up 90.5%, and apoptotic and dead cells amounted to 8.9%; relative to the ASO NC group, the miR-345 ASO group experienced an increase in the percentage of normal cells to 96.6%, and a decrease in the proportion of apoptotic and dead cells to 3.3%. *Compared to mimics NC, p<0.05; #Compared to ASO-NC, p<0.05.

## Data Availability

All data were obtained from open-source downloadable databases and did not involve permission. TCGA database was downloaded from the UCSC Xena website (https://xenabrowser.net/datapages/). EMT-related genes were downloaded from the dbEMT database (http://www.dbemt.bioinfo-minzhao.org/) and hypoxia-related genes were obtained from the MSigDB database (https://www.gseamsigdb.org/gsea/msigdb/cards/HALLMARK_HYPOXIA.html) (accessed on 12 November 2024).

## Abbreviations

HCC: Hepatocellular carcinoma
PVT1: Plasmacytoma variant translocation 1
TACE: Transarterial chemoembolization
EMT: Epithelial–mesenchymal transition
TME: Tumor microenvironment
HIF-1α: Hypoxia-inducible factor-1α
FOXM1: Forkhead box protein M1
lncRNA: Long noncoding RNA
TCGA: The Cancer Genome Atlas
ceRNA: Competing endogenous RNA
si-NC: Negative control
SD: Standard deviation
PFS: Progression-free survival time
ROC: Receiver operating characteristic
AUC: Area under the curve
CAF: Cancer-associated fibroblast
BMI: Body mass index
PIVKA: Protein induced by vitamin k absence or antagonist-II
AFP: Alpha-fetoprotein
CEA: Carcinoembryonic antigen
CA19-9: Cancer antigen 19-9
PT: Prothrombin time
INR: International normalized ratio
TT: Thrombin time
Fib: Fibrinogen
RBC: Red blood cell count
HGB: Hemoglobin
HCT: Hematocrit
MCV: Mean corpuscular volume
MCH: Mean corpuscular hemoglobin
MCHC: Mean corpuscular hemoglobin concentration
RDW: Red cell distribution width
PLT: Platelet count
WBC: White blood cell count
BASO: Basophils percentage
TB: Total bilirubin
DB: Direct bilirubin
IB: Indirect bilirubin
ALT: Alanine aminotransferase
AST: Aspartate aminotransferase
ALP: Alkaline phosphatase
GGT: Gamma-glutamyl transferase
CK: Creatine kinase
LDH: Lactate dehydrogenase
HBDH: Hydroxybutyrate dehydrogenase
TBA: Total bile acids
TP: Total protein
eGFR: Estimated Glomerular Filtration Rate
UA: Uric acid
TC: Total cholesterol
HDL-C: High-Density Lipoprotein Cholesterol
LDL-C: Low-density lipoprotein cholesterol

## Supplementary Materials

The supplementary material is available online at https://doi.org/10.32604/or.2024.056708.
