# Supplementary figures and images for: Hypoxia-induced exosomal lncRNA-PVT1 as a biomarker and mediator of EMT in hepatocellular carcinoma
Source: Oncol Res. 2025 May 29;33(6):1405–21. doi: 10.32604/or.2024.056708 (PMC12144658; doi:10.32604/or.2024.056708)

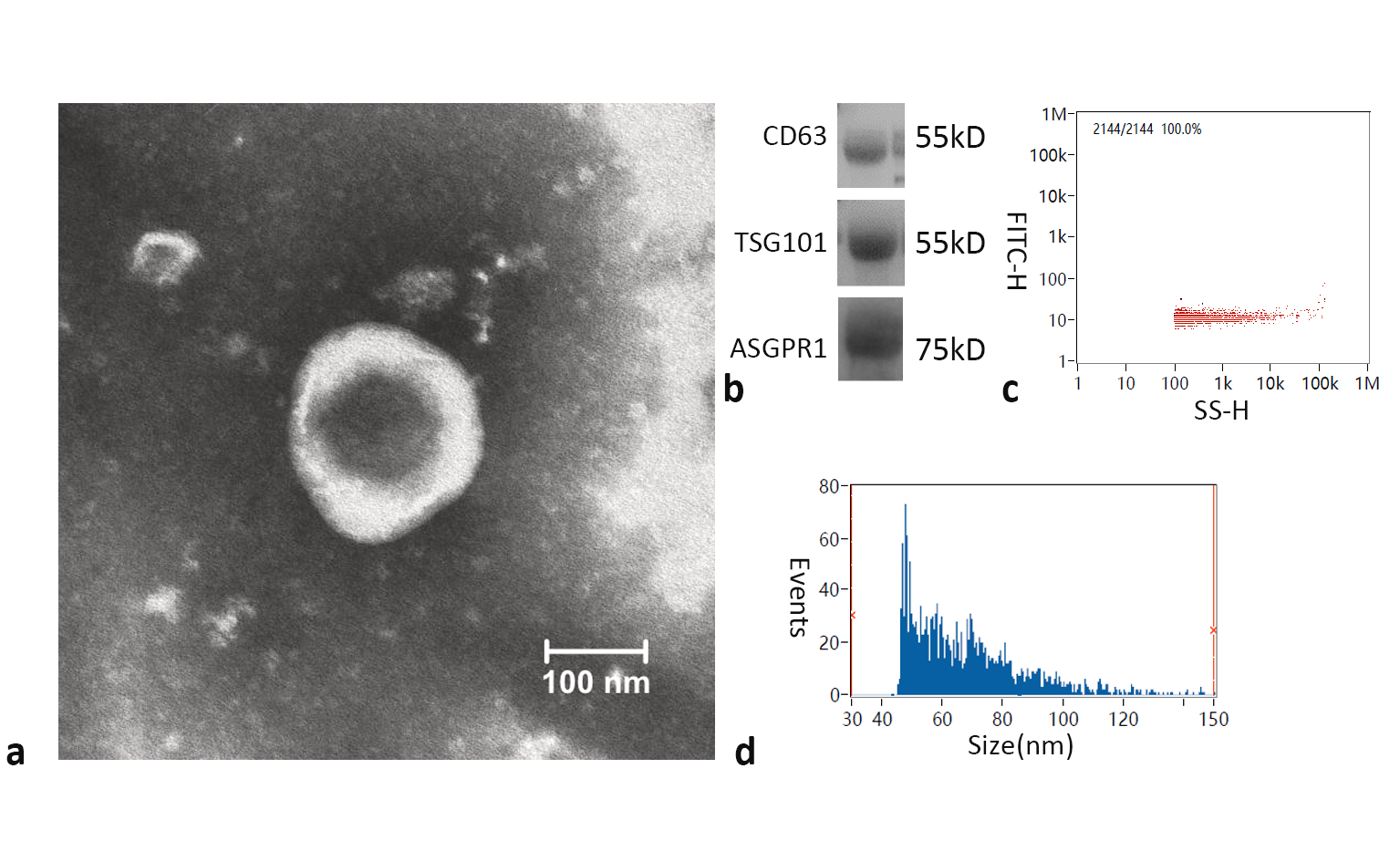

Supplement: Figure S1 [file OncolRes-33-56708-s001.tif]

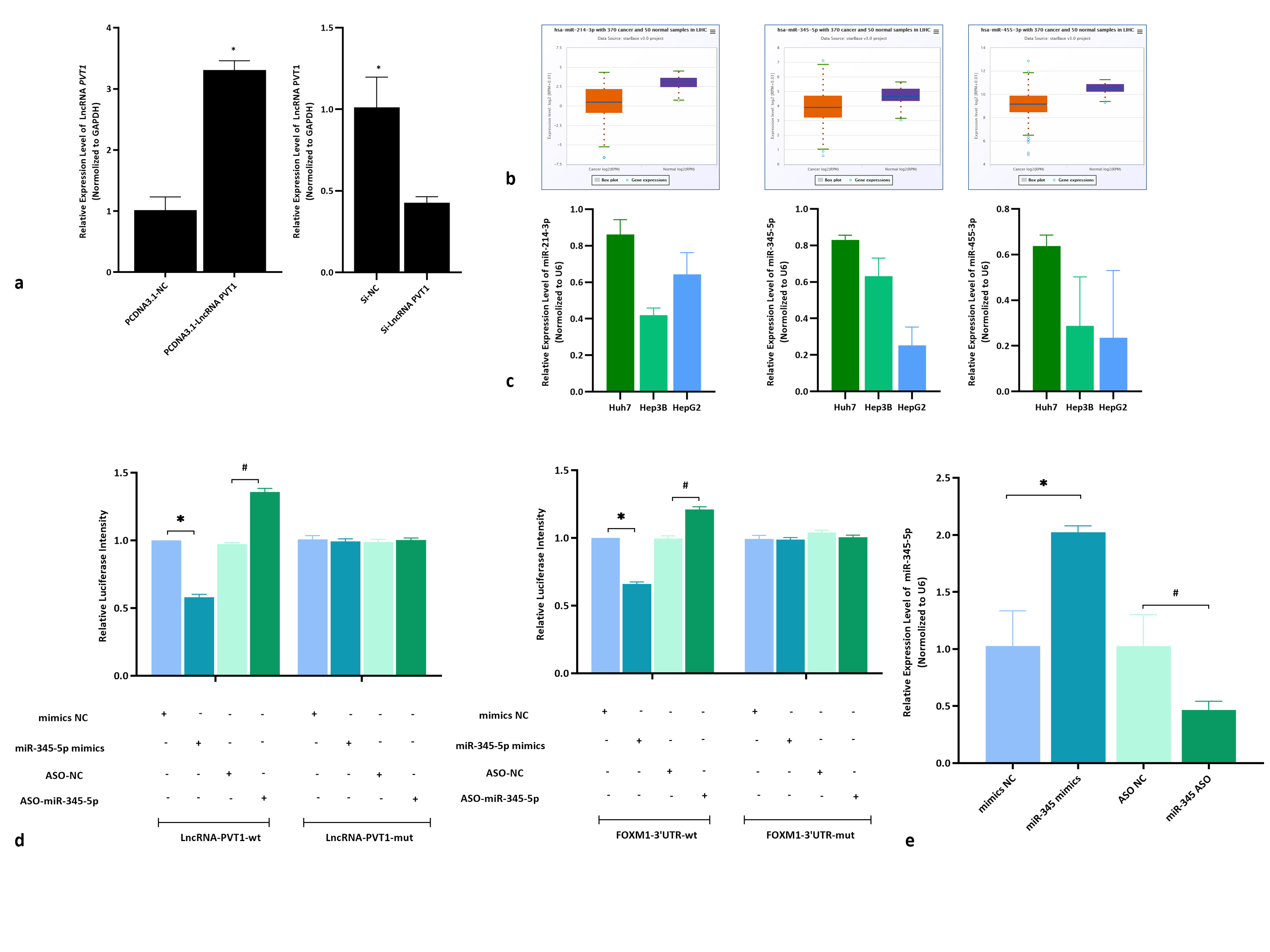

Supplement: Figure S3 [file OncolRes-33-56708-s003.tif]
