# Supplementary material for: Hypoxia-induced exosomal lncRNA-PVT1 as a biomarker and mediator of EMT in hepatocellular carcinoma
Source: Oncol Res. 2025 May 29;33(6):1405–21. doi: 10.32604/or.2024.056708 (PMC12144658; doi:10.32604/or.2024.056708)

**Figure Legends of Graphic Abstract** Potential role of plasma exosomal lncRNA-*PVT1* as a biomarker for prognostic evaluations after transarterial chemoembolization in hepatocellular carcinoma. Under hypoxia, the exosomes derived from highly invasive cells with elevated expression of lncRNA-*PVT1* target poorly invasive cells, promoting FoxM1 expression via a miRNA-345-5p-mediated competing endogenous mechanism, thereby facilitating their ability to undergo epithelial-mesenchymal transition (EMT).


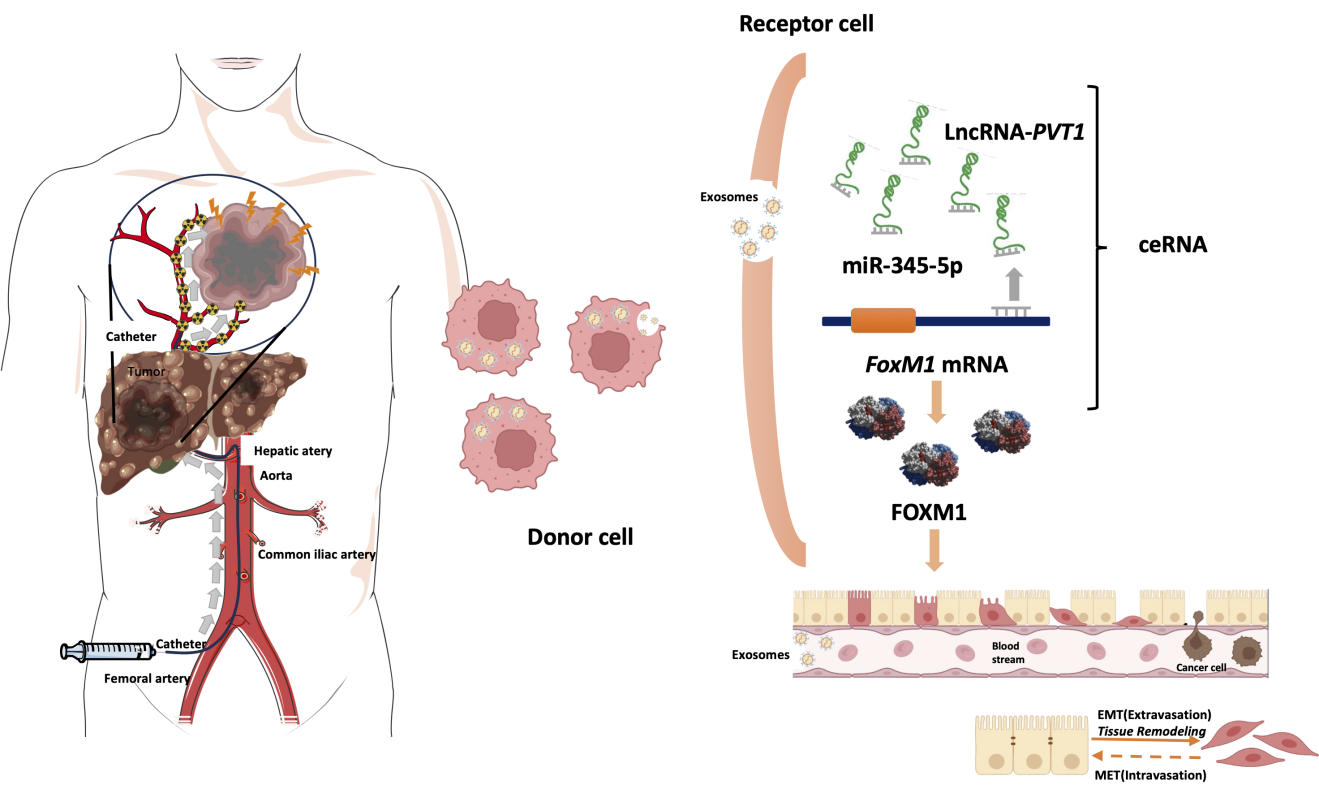

Supplement: Supplementary file 5 [file OncolRes-33-56708-s005.docx]
