# Supplementary material for: Hypoxia-induced exosomal lncRNA-PVT1 as a biomarker and mediator of EMT in hepatocellular carcinoma
Source: Oncol Res. 2025 May 29;33(6):1405–21. doi: 10.32604/or.2024.056708 (PMC12144658; doi:10.32604/or.2024.056708)
Supplement: Supplementary file 6 [file OncolRes-33-56708-s006.docx]

**Isolation and identification of plasma exosomes**

Isolation of plasma exosomes

1. Plasma was rapidly melted at 37℃, and then ≥ 1 mL of plasma was centrifuged at 3000 g and 4℃ for 15 min.
2. The supernatant (800 μL) was added with 200 μL of Exo Quick (SBI, Palo Alto, CA, USA), completely mixed by flicking the wall of the tube and inverting the tube, and allowed to stand at 4℃ for 2 h, followed by centrifugation at 1500 g and 4℃ for 30 min and removal of the supernatant.
3. The samples were resuspended with 500 μL of 1 × phosphate-buffered saline (PBS), centrifuged at 1500 g and 4°C for 5 min. After the supernatant was discarded, the white flocculent precipitate was plasma exosomes.

Identification of plasma exosomes

1. Transmission electron microscope observation of exosome morphology

Exosomes were resuspended with 100 μL of PBS, added dropwise onto a copper mesh (about 2 mm in diameter), and precipitated for 10 min, followed by the removal of the excess liquid with filter paper. Then, exosomes were precipitated for 1 min after 10 μL of 1% uranyl acetate was added dropwise on the copper mesh, and filter paper was used to absorb the excess liquid. The exosomes were dried at room temperature for 15 min, and their morphology was observed, imaged, and recorded under a transmission electron microscope at 80 kV.

1. Nanoparticle tracer analysis of exosome size and concentration

The exosomes were resuspended with 100 μL of PBS and then subjected to gradient dilution of 1,000-5,000 times to prevent the clogging of the sample needle by the samples. Standards were utilized to test the performance of the instrument. After the above test proved that the instrument was qualified, exosomes were put on the instrument for tracking and analyzing the Brownian motion of each particle. Subsequently, the hydrodynamic diameters and concentrations of exosomes were calculated with the Stokes-Einstein equation.

1. Immunoblotting to characterize exosomal membrane protein expression

Exosomal proteins and protein expression were detected with the same method as in the cellular experiments.

**LncRNA-PVT1 expression in plasma exosomes**

RNA extraction of plasma exosomes

1. The precipitate (exosomes) was added with 800 μL of QIAzol, triturated and mixed with a pipette until there was no visible precipitate, and allowed to stand for 5 min at room temperature;
2. The exosomes were added with 150 μL of chloroform and mixed through vigorous oscillation for approximately 20 s. The solution presented as a milky white slurry, which was left to stand for 2 min at room temperature and then centrifuged at 12,000 g and 4°C for 15 min;
3. The supernatant was transferred to a 1.5 mL clean microcentrifuge (Eppendorf [EP]) tube, added with 900 μL of absolute alcohol, and triturated and mixed with a pipette (be careful to avoid air bubbles as much as possible). After the samples were mixed again through gentle inversion, all products were transferred to the column (the amount of the products was ensured to be ≤ 700 μL) and centrifuged at 12,000 rpm for 15 s at room temperature, with the waste liquid discarded (if the amount of the products was > 700 μL, the step was repeated, and the products continued to be transferred to the column and centrifuged);
4. The column was added with 700 μL of buffer RWT and centrifuged at 12,000 rpm for 20 s at room temperature, followed by the discarding of the waste liquid in the elution column;
5. The column was added with 500 μL of buffer RPE and centrifuged at 12,000 g for 15 s at room temperature, with the waste liquid removed;
6. The column was added with 500 μL of buffer RPE and centrifuged at 12,000 g for 2 min at room temperature, with the waste liquid discarded;
7. The column was replaced with a new elution column (be careful not to be contaminated with the waste liquid). After uncapping, the column was centrifuged at room temperature for 5 min at 14,000 rpm, and the residual waste liquid was discarded;
8. The product was transferred to a 1.5 mL clean EP tube, added with 15 μL of enzyme-free water, and centrifuged at 14,000 rpm for 1 min at room temperature.
9. Real-time fluorescence quantitative polymerase chain reaction (PCR) was performed for the amplification of the target gene. The primer sequences of lncRNA-PVT1 are listed in Table 1. The present study involved an in vivo hypoxic environment. Accordingly, β-actin was used as an internal reference to prevent the influence of hypoxia on the expression of housekeeping genes.

**Table 1 The primer sequences of lncRNA** **PVT1 for PCR**

| Targets | | Company |
| --- | --- | --- |
| LncRNA-PVT1 | S: CCAGCACCTGCCTTATCCAA  AS: GAGTCCAGTGATGCTTCCATAGC | Sangon Biotech |
| β-actin | S: CTGGAACGGTGAAGGTGACA  AS: CGGCCACATTGTGAACTTTG |  |

Reverse transcription of plasma exosomal RNA

- 1. Preparation of the reaction system for reverse transcription.

Reverse transcription was conducted with a miRNeasy Serum/Plasma kit (Qiagen, Valencia, CA, USA). The reaction system is shown in Table 2.

**Table 2 The reaction system for reverse transcription**

| Compositions | Volume (μL) |
| --- | --- |
| gDNA Removal Mix | 2 |
| Internal Control RNA | 1 |
| Reverse Transcription Enzyme | 1 |
| Reverse Transcription Mix | 4 |
| Total RNA | 12 |
| Total volume | 20 |

The compositions were mixed thoroughly and reacted at 25°C for 3 min, 5°C for 10 min, and 85°C for 5 min.

- 1. Amplification of primer sequences of lncRNA-PVT1 and β-actin with fluorescence quantitative PCR

A QuantiNova SYBR-Green PCR Kit was used for real-time quantitative polymerase chain reaction. The reaction system is presented in Table 3.

**The reaction system for quantitative PCR**

| Compositions | Volume (μL) |
| --- | --- |
| 2X SYBR Green PCR Master Mix | 12 |
| Sense Primer (5 pmol/μL) | 1 |
| Antisense Primer (5 pmol/μL) | 1 |
| Enzyme-free water | 4 |
| Templet (cDNA obtained by reverse transcription was diluted 10-fold) | 4 |
| Total volume | 20 |

The compositions were mixed well and put on the machine after transient centrifugation, and the program was set as follows: pre-denaturation at 95°C for 10 min and 45 cycles of denaturation at 95°C for 15 s and annealing at 60°C for 32 s, followed by reaction at 95°C for 10 s, at 65°C for 60 s, and at 97°C for 1 s. Finally, the thermal melting curve was obtained.

- 1. Data processing The expression of lncRNA-PVT1 was calculated with the 2^-ΔΔCt^ method, and the formula was as follows:

ΔΔCt = (Ct1 - Ct2) - (Ct3 - Ct4)

Ct1: The critical cycle number of the gene to be tested (lncRNA-PVT1) in postoperative samples

Ct2: The critical cyclic number of the housekeeping gene (β-actin) in postoperative samples

Ct3: The critical cycle number of the gene to be tested (lncRNA-PVT1) in preoperative samples

Ct4: The critical cyclic number of the housekeeping gene (β-actin) in preoperative samples
