# Supplementary material for: Hypoxia-induced exosomal lncRNA-PVT1 as a biomarker and mediator of EMT in hepatocellular carcinoma
Source: Oncol Res. 2025 May 29;33(6):1405–21. doi: 10.32604/or.2024.056708 (PMC12144658; doi:10.32604/or.2024.056708)
Supplement: Supplementary file 7 [file OncolRes-33-56708-s007.docx]

***Hypoxia-Induced Exosomal lncRNA-PVT1 as a Biomarker and Mediator of EMT in Hepatocellular Carcinoma***

**Supplementary Table S1. The inclusion and exclusion criteria of the participants**

| **The inclusion criteria** |
| --- |
| 1. Age between 18-70 years old. 2. Liver function classified as Child-Pugh grade A or B. 3. Eastern Cooperative Oncology Group (ECOG) score of 0-2 points. 4. Expected survival period more than 3 months. 5. Tumor conditions:    1. HCC patients with clinical stages IIb and IIIa.    2. Patients with stage IIIb HCC, some with extrahepatic metastasis, who are expected to control intrahepatic tumor growth benefiting from TACE surgery.    3. If it's massive hepatocellular carcinoma, the volume of the tumor is less than 70% of the entire liver.    4. Patients with recurrence after hepatectomy for HCC.    5. Patients with recurrence after liver transplantation for HCC.    6. Patients with rupture and bleeding of HCC. |
| **The exclusion criteria** |
| 1. Patients with severe liver function damage, i.e., classified as Child-Pugh grade C. 2. Serious coagulation dysfunction that cannot be corrected after treatment. 3. The tumor embolus completely occluded the main portal vein with little collateral circulation formed. 4. Diffuse tumors with an expected survival period of less than 3 months. 5. ECOG score greater than 2, with cachexia or multiple organ failure. 6. Serious allergy to iodinated contrast agent. |

**Supplementary Table S2. Isolation and identification of exosomes**

| **Isolation** | |
| --- | --- |
| **plasma exosomes** | **exosomes from Cell Culture Medium** |
| 1. The plasma was quickly thawed at 37℃, followed by centrifugation at 3000 g and 4℃ for 15 min. 2. An 800 μL aliquot of the supernatant was collected and mixed with 200 μL of ExoTM Quick reagent. After thorough mixing, the solution was incubated at 4℃ for 2 h. Subsequently, it was centrifuged at 1500 g and 4℃ for 30 min, with the remaining supernatant discarded. 3. The pellet was resuspended in 500 μL of 1× PBS and then centrifuged at 1500 g and 4℃ for 5 min. The supernatant was removed, leaving behind a white flocculent precipitate that comprised the plasma exosomes. | 1. Cell culture media was collected and centrifuged at 3000g, room temperature for 20 min to remove cells and debris. 2. The remaining solution was diluted 1:1 with PBS, filtered through a 0.22 μmbacterial sieve, and then transferred to a new tube. 3. It was centrifuged at 15,000 g, 4℃ for 30 min, and the supernatant was diluted with PBS. 4. Ultracentrifugation at 150,000 g, 4℃ for 70 min, then supernatant was discarded. 5. Ultracentrifugation the remaining content washed with PBS again at 150,000 g, 4℃ for 70 min; the pellet was resuspended in 200 μL PBS. |
| **Identification** | |
| 1. Observation of Exosome Morphology by Transmission Electron Microscopy 2. Analysis of Exosome Size and Concentration by Nanoflow Cytometry 3. Identification of Exosomal Membrane Protein Expression by Western Blotting | |

**Supplementary Table S3. The sequence of the siRNAs for LncRNA-*PVT1***

| Position | 1643 | 372 | 878 |
| --- | --- | --- | --- |
| SS Sequence | GCAGCUUAUUAUAGACUUAUA | GGCACAUUUCAGGAUACUAAA | CCGAGUAGCUGGUAUUACAGG |
| AS Sequence | UAAGUCUAUAAUAAGCUGCAA | UAGUAUCCUGAAAUGUGCCGG | UGUAAUACCAGCUACUCGGGA |

**Supplementary Table S4. The sequence for dual-luciferase reporter assay**

| Gene | Sequence |
| --- | --- |
| LncRNA-PVT1 wt | 5’-caaCUUUUGG- UA-GAGUCAGc-3’ |
| LncRNA-PVT1 mut | 5’ -caa AUUCUAC- CG -ACAAGGAc-3’ |
| FoxM1 3'UTR wt | 5’- cuccccguguuuccaAGUCAGc- 3’ |
| miR-345-5p | 3’- cucgggaccugauccUCAGUCg- 5’ |
| FoxM1 3 UTR mut | 5’ – cuccccguguuuccaCAGACAc- 3’ |

**Supplementary Table S5. The sequences of miRNA mimics**

| mimics NC | SS Sequence | 5’-UCACAACCUCCUAGAAAGAGUAGA-3’ |
| --- | --- | --- |
|  | AS Sequence | 5’-UCUACUCUUUCUAGGAGGUUGUGA-3’ |
| miR-345-5p mimcs | SS Sequence | 5’-GCUGACUCCUAGUCCAGGGCUC-3’ |
|  | AS Sequence | 5’-CGACUGAGGAUCAGGUCCCGAG-3’ |

**Supplementary Table S6. The reaction system for reverse transcription and quantitative PCR**

| reverse transcription | |
| --- | --- |
| Compositions | Volume (μL) |
| gDNA Removal Mix | 2 |
| Internal Control RNA | 1 |
| Reverse Transcription Enzyme | 1 |
| Reverse Transcription Mix | 4 |
| Total RNA | 12 |
| Total volume | 20 |
| quantitative PCR | |
| Compositions | Volume (μL) |
| 2X SYBR Green PCR Master Mix | 10 |
| Sense Primer (5 pmol/μL) | 1 |
| Antisense Primer (5 pmol/μL) | 1 |
| Enzyme-free water | 4 |
| Templet (cDNA obtained by reverse transcription was diluted 10-fold) | 4 |
| Total volume | 20 |

**Supplementary Table S7. The primer sequences of lncRNA and mRNAs**

| Targets |  | Companies |
| --- | --- | --- |
| LncRNA-PVT1 | S: CCAGCACCTGCCTTATCCAA  AS: GAGTCCAGTGATGCTTCCATAGC | Sangon Biotech, Shanghai, China |
| β-actin | S: CTGGAACGGTGAAGGTGACA  AS: CGGCCACATTGTGAACTTTG |  |
| FoxM1 | S: ACCTTCTAGAATTCCTGAGCTACAGTAGAGC  AS: CCTATCTAGAGTCCACCTTCGCTTTTATTGAG |  |
| HIF-1α | S: CTCAGAATGAAGTGTACCCTAA  AS: CAAATCAGCACCAAGCAG |  |
| GAPDH | SBH1220545-200 | 249920, Qiagen, Valencia, CA, USA |
| miR-214-3p | F: ACAGCAGGCACAGACAGGCAGU  R: UGCCUGUCUGUGCCUGCUGUUU | Sangon Biotech, Shanghai, China |
| miR-345-5p | F: GAGGCGATCGCGTTGTGGAGAGAGGGAGGT  R: CTAGCGGCCGCGTTTCTGAAGCTCAACTATGGA |  |
| miR-455-3p | F: GAACTGCAGTCCATGGGCATA  R: GCAGGGTCCGAGGTATTC |  |
| U6 | F: TGCGGGTGCTCGCTTCGGCAGC  R: CCAGTGCAGGGTCCGAGGT |  |

**Supplementary Table S8. WB and IHC primary antibodies dilution ratios**

| primary antibodies | catalog number | manufacturer | dilution ratios |
| --- | --- | --- | --- |
| Anti-E-cadherin antibody | ab1416 | Abcam, Cambridge, UK | 1:50 |
| Anti-N-cadherin antibody | ab245117 |  | 1:1000 |
| Anti-Vimentin antibody | ab92547 |  | 1:1000 |
| Anti-GAPDH antibody | HRP-60004 | proteintech, Wuhan, China | 1:1000 |
| Anti-FOXM1 antibody | ab207298 | Abcam, Cambridge, UK | 1:1000 for WB  1:250 for IHC |
| Anti-Ki67 antibody | 28074-1-AP | proteintech, Wuhan, China | 1:8000 |
| Anti-MMP9 antibody | 10375-2-AP |  | 1:50 |

**Supplementary Table S9. 15 candidates’ mRNAs**

| Source Id | NCBI (Entrez) Gene Id | Gene Symbol |
| --- | --- | --- |
| 332 | 332 | BIRC5 |
| 29923 | 29923 | CTHRC1 |
| 1894 | 1894 | ECT2 |
| 2305 | 2305 | FOXM1 |
| 2934 | 2934 | GSN |
| 3486 | 3486 | IGFBP3 |
| 81625 | 3958 | LGALS3 |
| 4017 | 4017 | LOXL2 |
| 4605 | 4605 | MYBL2 |
| 10460 | 10460 | TACC3 |
| 7159 | 7159 | TP53BP2 |
| 7298 | 7298 | TYMS |
| NM_000125 | 2099 | ESR1 |
| NM_003955 | 9021 | SOCS3 |
| NM_007315 | 6772 | STAT1 |

**Supplementary Table S10. Comparison of Laboratory Results Before and After TACE**

| Tests | Before TACE | After TACE | *p*-value |
| --- | --- | --- | --- |
| RBC (10^12^/L) | 4.34±0.82 | 4.24±0.77 | 0.019 |
| HGB (g/L) | 132±24.6 | 130±25.9 | 0.021 |
| HCT (L/L) | 0.40±0.07 | 0.39±0.07 | 0.060 |
| MCV (fL) | 94.5±8.03 | 93.3±8.04 | <0.001 |
| MCH | 30.7±3.15 | 30.7±3.23 | 0.894 |
| MCHC (pg) | 324±12.74 | 328±12.54 | <0.001 |
| RDW (fL) | 50.6±7.22 | 53.4±44.8 | 0.537 |
| PLT (10^9^/L) | 120±78 | 114±76 | 0.150 |
| WBC (10^9^/L) | 5.03±3.18 | 7.38±3.42 | <0.001 |
| NEUT (%) | 60.4±12.45 | 76.56±9.35 | <0.001 |
| LYMPH (%) | 27.2±10.86 | 13.7±7.02 | <0.001 |
| MONO (%) | 8.4 (7.1, 10.2) | 7.4 (6.2, 9.2) | 0.985 |
| EO (%) | 2.1 (1.3, 4.2) | 1.3 (0.4, 2.5) | <0.001 |
| BASO (%) | 0.5 (0.3, 0.8) | 0.3 (0.2,0.4) | <0.001 |
| TB (umol/L) | 18.2+8.41 | 25.9±12.20 | <0.001 |
| DB (umol/L) | 6.6±4 | 9.8±5.59 | <0.001 |
| IB (umol/L) | 11.6±5.48 | 16.1±7.78 | <0.001 |
| TBA (umol/L) | 12.2 (4.95, 26.6) | 7.9 (3.78, 22.5) | 0.021 |
| ALT (IU/L) | 28 (21, 42) | 54 (31, 117) | <0.001 |
| AST (IU/L) | 36 (27, 51) | 104 (41, 215) | <0.001 |
| AST/ALT | 1.32 (1.04, 1.78) | 1.46 (1.11, 2.23) | 0.001 |
| TP (g/L) | 69.2±6.13 | 67.6±6.96 | 0.020 |
| ALB (g/L) | 39.1±4.29 | 38.2±4.66 | 0.036 |
| GLB (g/L) | 30.2±6.22 | 29.6±6.19 | 0.126 |
| ALB/GLB | 1.36±0.37 | 1.36±0.36 | 0.908 |
| ALP (IU/L) | 143±89 | 150±98 | 0.172 |
| GGT (IU/L) | 87 (52, 184) | 95 (45, 190) | 0.432 |
| CK (IU/L) | 66 (42, 96) | 64 (45, 92) | 0.059 |
| LDH (IU/L) | 196 (164, 245) | 301 (210, 500) | <0.001 |
| HBDH (IU/L) | 144(119,178) | 204 (148, 237) | 0.001 |
| GLU (mmol/L) | 5.15±0.92 | 6.27±2.61 | <0.001 |
| UREA (mmol/L) | 4.7±1.61 | 4.35±1.56 | 0.012 |
| CREA (umol/L) | 74±20.3 | 73.6±21.40 | 0.325 |
| eGFR (ml/min/1.73m^2^) | 91.89±18.74 | 92.32±19.32 | 0.502 |
| Cys-c (mg/L) | 1.07±0.33 | 0.98±0.33 | <0.001 |
| UA (umol/L) | 332±106 | 290±102 | <0.001 |
| TG (mmol/L) | 1.05±0.48 | 1±0.37 | 0.222 |
| TC (mmol/L) | 3.75±0.85 | 3.45±0.83 | <0.001 |
| HDL-C (mmol/L) | 1.08±0.31 | 1.01±0.32 | <0.001 |
| LDL-C (mmol/L) | 2.21±0.65 | 2.04±0.67 | <0.001 |
